# The Role of SOX2 and SOX9 in Radioresistance and Tumor Recurrence

**DOI:** 10.3390/cancers16020439

**Published:** 2024-01-19

**Authors:** Silvia Barbosa, Natalia Koerich Laureano, Wahyu Wijaya Hadiwikarta, Fernanda Visioli, Mahnaz Bonrouhi, Kinga Pajdzik, Cristina Conde-Lopez, Christel Herold-Mende, Gustavo Eidt, Renan Langie, Marcelo Lazzaron Lamers, Fabian Stögbauer, Jochen Hess, Ina Kurth, Adriana Jou

**Affiliations:** 1 Division of Radiooncology/Radiobiology, German Cancer Research Center (DKFZ), 69120 Heidelberg, Germany; 2 Department of Otolaryngology, Head and Neck Surgery, University Hospital Heidelberg, 69120 Heidelberg, Germany; 3 Department of Morphological Sciences, Institute of Basic Health Science, Federal University of Rio Grande do Sul (UFRGS), Porto Alegre 90035-003, RS, Brazil; 4 Department of Oral Pathology, Faculty of Dental Sciences, Federal University of Rio Grande do Sul (UFRGS), Porto Alegre 90035-004, RS, Brazil; 5 German Cancer Consortium (DKTK), Core Center Heidelberg, 69120 Heidelberg, Germany; 6 Department of Neurosurgery, University Hospital Heidelberg, 69120 Heidelberg, Germany; 7 Tissue Bank of the National Center for Tumor Diseases (NCT) Heidelberg, Germany and Institute of Pathology, Heidelberg University Hospital, 69120 Heidelberg, Germany; 8 Institute of Pathology, School of Medicine, Technical University of Munich (TUM), 80337 Munich, Germany; 9 Heidelberg Institute of Radiation Oncology (HIRO), 69120 Heidelberg, Germany; 10 Molecular Mechanisms of Head and Neck Tumors, German Cancer Research Center (DKFZ), 69120 Heidelberg, Germany; 11 Faculty of Dentistry, Institute of Toxicology and Pharmacology, Pontifícial Catholic University of Rio Grande do Sul (PUCRS), Porto Alegre 90619-900, RS, Brazil

**Keywords:** SOX2, SOX9, HNSCC, HPV negative, gene set signature, metastasis, radiation treatment

## Abstract

**Simple Summary:**

Cancers of the head and neck are a diverse group of diseases with a wide range of clinical outcomes. In many patients, metastasis and resistance to radiotherapy are associated with a higher mortality rate. Our study showed that inverted expression of SOX2 and SOX9 differs between subtypes. We propose a gene set signature that can be used to stratify patients based on their expression pattern in to optimize treatment.

**Abstract:**

Head and neck squamous cell carcinoma (HNSCC) exhibits considerable variability in patient outcome. It has been reported that SOX2 plays a role in proliferation, tumor growth, drug resistance, and metastasis in a variety of cancer types. Additionally, SOX9 has been implicated in immune tolerance and treatment failures. SOX2 and SOX9 induce treatment failure by a molecular mechanism that has not yet been elucidated. This study explores the inverse association of SOX2/SOX9 and their distinct expression in tumors, influencing the tumor microenvironment and radiotherapy responses. Through public RNA sequencing data, human biopsy samples, and knockdown cellular models, we explored the effects of inverted SOX2 and SOX9 expression. We found that patients expressing SOX2^Low^SOX9^High^ showed decreased survival compared to SOX2^High^SOX9^Low^. A survival analysis of patients stratified by radiotherapy and human papillomavirus brings additional clinical relevance. We identified a gene set signature comprising newly discovered candidate genes resulting from inverted SOX2/SOX9 expression. Moreover, the TGF-β pathway emerges as a significant predicted contributor to the overexpression of these candidate genes. In vitro findings reveal that silencing SOX2 enhances tumor radioresistance, while SOX9 silencing enhances radiosensitivity. These discoveries lay the groundwork for further studies on the therapeutic potential of transcription factors in optimizing HNSCC treatment.

## 1. Introduction

Head and neck squamous cell carcinoma (HNSCC) is the sixth most common solid malignant tumor worldwide, with an estimated five-year survival rate of 66% in the last decades [1,2]. The most common malignancy of the oral cavity, pharynx and larynx, HNSCC comprises a range of tumors with different molecular, cellular and clinical characteristics [3,4]. Risk factors are tobacco, alcohol consumption, environmental exposure to carcinogens and viral infections [5]. The Human Papilloma Virus (HPV) status is an independent risk factor, and HPV-positive and HPV-negative tumors differ in molecular signatures, disease progression, and clinical response to treatment [6,7,8]. Apoptosis, survival, cell cycle, DNA replication, repair, immune response, transcription factors, epigenetic mechanisms, and tumor suppressors and oncogenes are among these molecular differences [7,9,10,11].

Dysregulation of SOX genes in cancer have been implicated in tumorigenesis, changes in the tumor microenvironment (TME), metastasis, and treatment resistance [12,13,14]. Among the SOX genes, the transcription factors (TFs) SOX2 and SOX9 have been pointed as important players in different types of cancer. In gastric cancer, SOX2 expression was correlated with tumor suppressor activity by modulating the WNT/β catenin pathway in a mouse model [15]. In breast cancer, SOX2 expression was linked with stemness properties which lead to hormone therapy resistance [16]. In lung cancer, stemness related to SOX2 has been linked to the release of cytokines in the TME, resulting in tumor cell plasticity and tolerance to therapy [17]. In HNSCC, low SOX2 expression was correlated with poor clinical prognosis and with an increased migration of the tumor cells [18]. Moreover, high SOX2 expression in HNSCC served as a good prognostic marker together with EpCAM and vimentin in patients treated with radio-chemotherapy [19]. In recent years, a number of reports have elucidated the involvement of SOX9 either as an oncogene or as a tumor suppressor gene [20]. In breast cancer, SOX9 expression has been linked to regulation of cancer stem cell properties, EMT, metastasis and poor clinical prognostic [21]. In non-small cell lung carcinoma (NSCLC), SOX9 contributes to tumor development and growth [22]. In gastric cancer, the SOX9 expression was associated with collagen type X alpha 1 (COL10A1) to promote migration and invasion of tumor cells [23]. A versatile role for SOX9 has been described. It can activate or repress transcription depending on the DNA target site, cofactor and cellular context to transcriptionally activate expression [24].

Lin et al., reported an epigenetic switch between SOX2 and SOX9 expression which modulates the plasticity of lung cancer cells. Tumor cells with reduced SOX2 levels show increased expression of mesenchymal markers, which is accompanied by the loss of morphological characteristics of epithelial cells and an increase in SOX9 expression [25]. Malladi et al., demonstrated the imbalance in SOX2/SOX9 expression as an essential factor for stem cell identity, pluripotency, immune surveillance, and metastasis. They have shown that the presence of latency-competent cancer cells in lung and breast tumors may be a mechanism that suppresses outgrowth, long-term survival, and maintenance of stemness property. The difference in SOX2/SOX9 expression and WNT signaling silencing enables tumor cells to enter a quiescent state and to evade the immune clearance by natural killer (NK) cells, finally resulting in propagation into metastasis-establishing cells [12]. More recently, Laughney et al. showed that the selective pressure of the immune surveillance can modulate the expression of SOX2 and SOX9 to enable tumor cell adaptation and immune escape. In a mouse model, they identified a quiescent stage in which tumor cells are undetected for an extended period. These findings point to increased SOX2 expression during tissue regeneration and the early phases of tumor development. On the other hand, high expression of SOX9 is present in proliferative, regenerative stages and inhibits the killing by NK cells [26]. The molecular mechanisms involved in SOX2/SOX9 regulation in HNSCC is still unknown. In this study, we aim to demonstrate that the association of two TFs has the potential to stratify patients using transcriptional datasets and patient-derived samples. Clinical parameters are crucial for enhancing survival accuracy. Additionally, we demonstrate, in a cellular model, the impact of radiotherapy on the expression patterns of SOX2 and SOX9. As an alternative approach for patient stratification, we propose a newly gene set signature which highlights the involvement of TGF-β signaling as predicted pathway acting in gene regulation and tumor response.

## 2. Materials and Methods

### 2.1. Expression and Clinical Data Sets

The RNA-seq count data of The Cancer Genome Atlas (TCGA) were downloaded from a public repository, the GDC portal (https://portal.gdc.cancer.gov accessed on 24 November 2021). The clinical and pathological data were downloaded from the cBioPortal (http://www.cbioportal.org accessed on 25 November 2021) upon selecting Head and Neck Study cohorts based on the TCGA PanCancer data set (TCGA-HNSCC, *n* = 530). The Heidelberg Center for Personalized Oncology (HIPO) HNC data set was used as an independent validation cohort (GSE117973) [27]. RNA sequencing data for the HNO223 cell lines have been uploaded to GEO and are available upon request.

### 2.2. Differential Gene Expression Analysis and Co-Expression

The analysis of differentially expressed genes (DEGs) was performed on TCGA-HNSCC data using R software (version 3.6.1; 5 July 2019) with the DESeq2 package from Bioconductor [28]. Data were ranked by inverse expression of SOX2 and SOX9 genes after normalization. Co-expressed candidate genes for either SOX2 or SOX9 were extracted from TCGA-HNSCC cohort data according to their Spearman correlation [29,30]. The files were loaded in R software and analyzed using packages: survminer, survival and ggplot2.

### 2.3. Images Analysis

Image acquisition was made using a slide scanner (Axioscan, Zeiss, Oberkochen, Germany). The whole slides were scanned at 20× magnification and the images were imported into QuPath software (version 0.3.2) following a pipeline of analyzes according to software documentation [31].

### 2.4. Cellular Culture and Gene Silencing

The human HNSCC cell line HNO223 was purchased from CSL (Cell Line Service GmbH, Eppelheim, Germany). The HNO223-Luci and HNO223-shSOX2 cell lines had their origin previously described [18]. HNO223-shSOX9, non-target and positive control cell lines were generated at the DKFZ Proteomics Core Facility using Dharmacon™ Inducible shRNA according to the manufacturer’s instruction (Cat. No. V3SH11255-02EG6662). The HNO223-Luci and HNO223-shSOX2 cell lines were kept under selection adding 60 μg/mL of Zeocin^TM^ (Invitrogen, Berlin, Germany) in DMEM complete medium. The HNO223-shSOX9 were treated with 1μg/mL of Doxycycline (Fischer Scientific, New Hampshire, EUA, Cat. No. ICN19895510) in DMEM complete medium. All cell lines were mycoplasma free, confirmed by the PCR-based detection using a mycoplasma detection kit (Thermo Fischer Scientific, Darmstadt, Germany). Cells were cultivated with Dulbecco’s Modified Eagle’s Medium (DMEM, Sigma-Aldrich, Darmstadt, Germany) supplemented with 10% fetal bovine serum (Invitrogen, Germany), penicillin-streptomycin 50 μg/mL (Invitrogen, Germany) and 2 M glutamine (Invitrogen, Germany). Cells were kept in humidified and sterile conditions with 5% CO_2_ at 37 °C.

### 2.5. Colony Formation Assay and Irradiation Assay

The three-dimensional (3D) colony formation assay, 96-well plates ultra-low attachment (Corning, Cat. No. 3474, Glendale, AZ, USA) with 150μL of a media-Matrigel mixture (1:1) containing 1.5 × 10^3^ cells per well were used. The plates were immediately submitted to a single dose of 2 Gy, 4 Gy, 6 Gy or 8 Gy (200 kV, 17.8 mA, 0.5 mm Cu filter, MultiRad Faxitron Precision) or multiple doses of 2 Gy for a period of 5 days. The control plates were omitted from irradiation (0 Gy).

After one overnight cultivation, 50 μL of media was carefully added into each well. The plates were kept at 37 °C and 5% CO_2_ in a humidified incubator for 10 days. Plates were scanned in a Nikon Eclipse Ti with 2× objective and colonies with a diameter higher than 50 μm were counted using QuPath software (version 0.3.2). Surviving fractions was calculated was previous described [32].

### 2.6. mRNA Expression Analysis

Total RNA was extracted by RNeasy Mini Kit (Qiagen), according to the manufacturer’s instructions. Reverse transcription was performed using the SuperScript^TM^ III Reverse Transcriptase Kit (Thermo Fischer Scientific) according to the manufacturer’s instructions. Quantitative real-time PCR was conducted on StepOnePlus (Applied Biosystems, Beverly, MA, USA) using SYBR^®^ Green Master Mix (Thermo Fisher Scientific) with 20 nM primers and 20 ng of cDNA. The relative gene expression was calculated by normalization to a housekeeping gene (GAPDH and ACTB) and ΔΔCT method. For primer sequences see Supplementary Materials.

### 2.7. RNA Sequencing

Total RNA was extracted from 10^6^ cells using Trizol (Life Technologies, Carlsbad, CA, USA) according to the manufacturer’s instructions. DNA treatment was performed using DNase I digestion (Qiagen). RNA was eluted using nuclease-free water (Thermo Fisher Scientific) and the concentration measured with Qubit Kit (Thermo Fisher Scientific). Samples with RIN (RNA integrity number) > 9 were processed. Sequencing libraries were prepared using the Illumina TruSeq mRNA stranded Kit following the manufacturer’s instructions. Briefly, mRNA was purified from 200 ng of total RNA using oligo(dT) beads. Then poly(A)+ RNA was fragmented to 150 bp and converted to cDNA. The cDNA fragments were then end-repaired, adenylated on the 3′ end, adapter ligated and amplified with 15 cycles of PCR. The final libraries were validated using Qubit (Invitrogen, Germany) and TapeStation (Agilent Technologies, Santa Clara, CA, USA). 2× 50 bp paired-end sequencing was performed on the Illumina NovaSeq 6000 according to the manufacturer’s protocol. At least 34 Mio. reads per sample were generated. Raw sequencing data were processed following the DKFZ/ODCF RNA-seq workflow (https://github.com/DKFZ-ODCF/RNAseqWorkflow) accessed on 27 December 2022. The final counts were used for differential gene expression analysis by applying DESeq2 package. The ranking of genes and descriptive visualization was performed by applying the lfcShrink function (apeglm method) for effect size shrinkage was previous described [33].

### 2.8. Western Blotting Analysis

Whole cell protein lysate was extracted using Radio-Immunoprecipitation Assay (RIPA) buffer (Sigma Aldrich, St. Louis, MO, USA) plus protease and phosphatase inhibitor cocktail (Sigma-Aldrich). The SDS-PAGE consisting of 12% Acrylamide/Bis gel was used with equal amounts of protein samples. Membranes were incubated for one overnight with the primary antibodies against SOX2 (α-rabbit; 1:1000; Cell Signaling—D6D9, Danvers, MA, USA) or SOX9 (α-rabbit; 1:1000; Cell Signaling—D8G8H). The secondary antibody horseradish radish peroxidase (HRP; Cell Signaling—7074S) was incubated for 1 h at room temperature. Subsequently, signal was then detected by ImageQuant LAS500 system (GE Healthcare Life Science, Chicago, IL, USA) using LumiGLO^®^ chemiluminescence solution (Cell Signaling—7003S). Beta-Actin (alpha-rabbit; 1:1000; Cell Signaling—D6A8), and/or GAPDH (α-rabbit; 1:1000; Cell Signaling—D16H11), were used as loading for both quantity and quality control of protein lysates.

### 2.9. Immunohistochemistry (IHC)

Samples from the HIPO-HNC cohort (GSE117973) were provided by the tissue bank of the National Center for Tumor Diseases (NCT, Heidelberg, Germany) in accordance with the regulations of the tissue bank and the approval of the Ethics Committee of the Heidelberg University Hospital (under protocols S-206/2011, S-220/2016 and S-786/2021). IHC staining was performed using anti-SOX2 (Cell Signaling, D6D9) or anti-SOX9 (Cell Signaling, D8G8H or ABCAM, 76997) antibodies as described previously [18]. The whole stained slices were scanned using a slide scanner (Axioscan, Zeiss, Oberkochen, Germany) with a 20× magnification objective. The immune-reactivity score (IRS) was computed as a product of the staining intensity (1: mild, 2: moderate and 3: strong) and the percentage of positively stained tumor cells ranging from 0–100%. The final immune-reactivity score (IRS) was calculated as described [18].

### 2.10. Statistical Analysis

Statistical analyses were performed using GraphPad Prism software (version 9.3.1) and R software (version 4.1.1). *p*-values were represented as: ns, not significant (*p* > 0.05); * *p* ≤ 0.05; ** *p* ≤ 0.01 and *** *p* ≤ 0.001. Data are presented as means values ± SEM in at least 3 biological and technical replicates. Statistical tests are indicated according to each analysis.

## 3. Results

### 3.1. Inverse SOX2 and SOX9 Expression Correlates with Disease Specific Survival in HNSCC

SOX2 and SOX9 are often deregulated in cancer and a dual role was described for both TFs [18,19,20]. To explore the hypothesis that these stem cell TFs influence the prognosis of HNSCC patients, computational data analysis at mRNA level of the TCGA-HNSCC cohort was performed. To first investigate whether SOX2 and SOX9 predict survival, Kaplan-Meier curves were plotted for each individual TF, and showed an unfavorable disease-specific survival (DSS) for HNSCC patients with low SOX2 (Supplementary Figure S1A) and high SOX9 (Supplementary Figure S1B) expression. Following these observations, Spearman’s correlation revealed a modest inverse association between both TFs on mRNA expression level (Supplementary Figure S1C). To explore better these observations a second transcriptome data set of an independent HNSCC cohort (HIPO-HNC, GSE117973) was explored. As previous observed, unfavorable DSS for HIPO-HNC patients presenting low SOX2 (Supplementary Figure S1D) and high SOX9 (Supplementary Figure S1E) gene expression was confirmed. Spearman’s correlation demonstrated a trend towards an inverse correlation between SOX2 and SOX9 transcript levels, which did not reach statistical significance (Supplementary Figure S1F).

**Figure 1:**
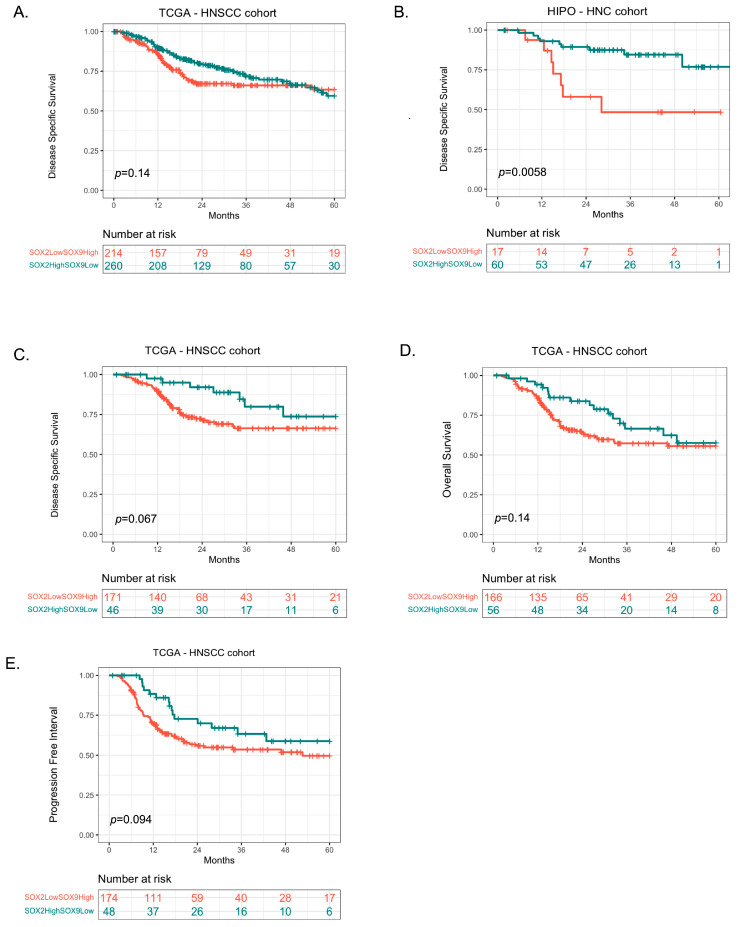
Survival Characteristics of SOX2 and SOX9 Expression in HNSCC Patient Cohorts. (**A**). Disease Specific Survival of TCGA-HNSCC patients grouped stratified according to inverse SOX2 and SOX9 expression (*n* = 474). (**B**). Disease specific survival of HIPO-HNC patients grouped according to inverse SOX2 and SOX9 expression (*n* = 77). (**C**). Disease specific survival of TCGA-HNSCC patients grouped according to inverse SOX2 and SOX9 expression and stratified for HPV negative and Radiation treatment (*n* = 217). (**D**). Overall Survival of TCGA-HNSCC patients grouped according to the ratio between SOX2 and SOX9 expression and stratified for HPV negative and Radiation treatment (*n* = 222). (**E**). Progression Free Interval of TCGA-HNSCC patients grouped according to the ratio between SOX2 and SOX9 expression and stratified for HPV negative and Radiation treatment (*n* = 222). Missing values were removed from the analysis. Kaplan Meier analysis was performed in R software using the package survival and ggplot2.

With the focus on associate the inverse expression of both TFs the ratio between SOX2 and SOX9 was calculated after gene normalization for each patient of the cohorts and Maxstat algorithm was applied to define a cut-off point for patient stratification into either a group of low SOX2 and high SOX9 expression (SOX2^Low^SOX9^High^) or a group of high SOX2 and low SOX9 expression (SOX2^High^SOX9^Low^). In TCGA-HNSCC, clinical characteristics were found to differ between groups with opposite expression levels of SOX2 and SOX9, with significance observed for gender, HPV status, tumor location, and radiation treatment (Table 1). However, Kaplan Meier survival analysis confirmed a trend towards unfavorable DSS for patients with SOX2^Low^SOX9^High^, which did not reach statistical significance (Figure 1A). For the HIPO-HNSC cohort the group of patients with SOX2^Low^SOX9^High^ confirmed a worse survival as compared to the opposite group (Figure 1B). We further tested whether the association with clinical parameters add accuracy to the survival probability. Survival curve was calculated for TCGA-HNSCC cohort focus on HPV negative status with radiation treatment. Indeed, we could observe that the HPV negative HNSCC patient group with SOX2^Low^SOX9^High^ expression and with radiation treatment had a reduced survival probability when compared to the group with SOX2^High^SOX9^Low^ expression that was most prominent for DSS (Figure 1C). Overall Survival (OS) and Progression Free Interval (PFI) showed the trend toward a reduced survival probability which did not reach statistical significance. Taken together, it was possible to observe that patients with SOX2^Low^SOX9^High^ expression present a trend to reduced survival especially those with HPV negative status and undergo to radiation treatment.

### 3.2. Gene signature Based in the Inverse Expression of SOX2 and SOX9

**Figure 2:**
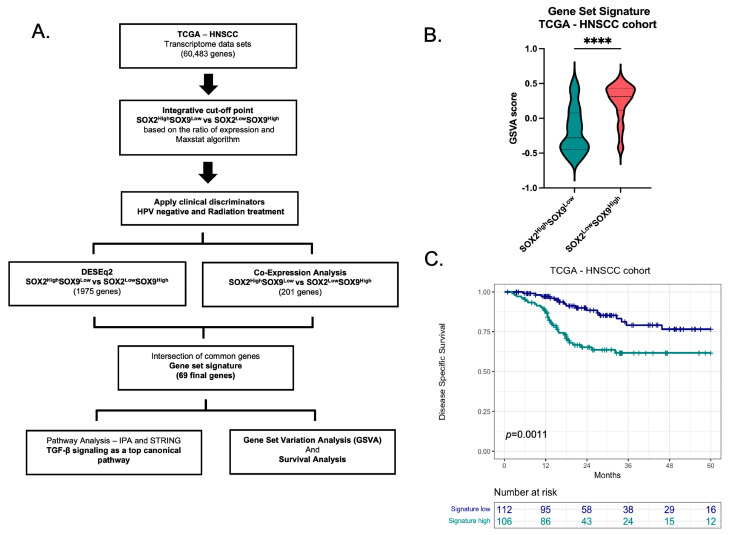
Signature Gene Set based on the inverse SOX2 and SOX9 expression. (**A**). Schematic representation for identify the genes set signature. (**B**). GSVA score in SOX2^High^SOX9^Low^ and SOX2^Low^SOX9^High^ for HPV negative status patients treated with radiotherapy in TCGA-HNSCC (*n* = 222). (**C**). Kaplan Meier analysis in HPV negative status patients treated with radiotherapy in TCGA-HNSCC (*n* = 222). Statistical analysis was performed in Prism 9.0 or R software. Missing data was removed from the analysis. **** *p* < 0.0001.

Two distinct bioinformatic approaches were employed to investigate whether the inverse SOX2 and SOX9 expression may predict potential candidate genes in HPV-negative patients treated with radiation. Initially, a co-expression analysis was conducted to identify potential candidate genes in HPV-negative HNSCC patients from the TCGA cohort who underwent radiotherapy. A moderated Spearman’s correlation cut-off point of 0.2 was applied to delineate two gene sets based on patient groups: SOX2^Low^SOX9^High^ and SOX2^High^SOX9^Low^. It is noteworthy that genes correlated with SOX9 exhibited a narrower range of Spearman’s correlations compared to genes correlated with SOX2. The intersection of these two gene sets revealed a set of 201 genes associated with inverse SOX2 and SOX9 expression (Supplementary Table S1). In addition, analysis of Differentially Expressed Genes (DEGs) between SOX2^Low^SOX9^High^ versus SOX2^High^SOX9^Low^ in TCGA-HNSCC in patients in HPV negative with radiotherapy revealed that 1975 genes were being affected by this inverse regulation. To establish a distinctive gene set signature, an intersection analysis was conducted between the co-expressed genes and the DEGs, resulting in a final set of 69 genes being identified (Figure 2A and Supplementary Table S2). Thus, to explore the clinical relevance of this gene set signature, a gene set variation analysis (GSVA) was calculated and demonstrated that the group of patients with SOX2^Low^SOX9^High^ expression presented high score for the newly identified gene set signature (Figure 2B). In addition, the high expression of the gene set signature was observed in the group with SOX2^Low^SOX9^High^, which also demonstrated a reduced survival probability when compared to the group with SOX2^High^SOX9^Low^ expression (Figure 2C).

### 3.3. TGF-β Signaling as One of the Top Canonical Pathways Regulated by Inverse SOX2/SOX9 Expression

**Figure 3:**
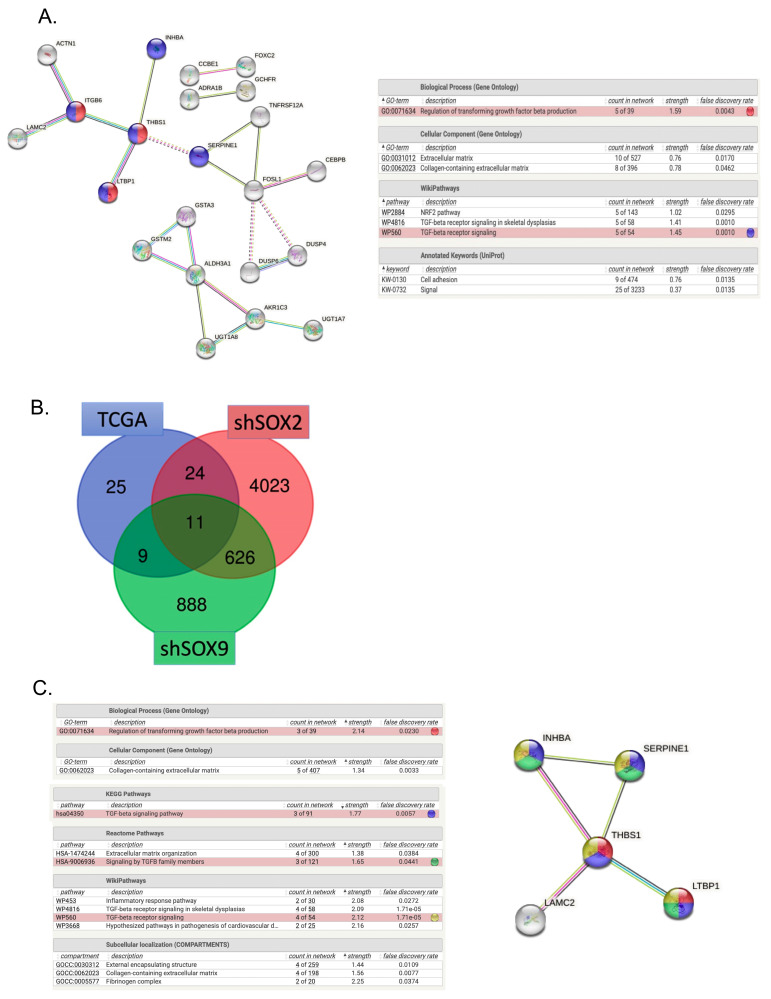
Molecular and Cellular Functions Affected by the Gene Signature. (**A**). Functional analysis of the predicted gene signature for Gene Ontology (GO) and biological pathways. The lines represent protein-protein interactions mapped by STRING. The blue nodes are genes connected to TGF-β signaling and the red nodes represent genes involved in the regulation of the TGF-β production. (**B**). Venn diagram intercepting the predicted gene signature by TCGA patients, differential gene expression for HNO223 cell lines silenced for SOX2 or SOX9. The total set of common genes consisted of 11 genes. (**C**). Analysis of the intersection predicted gene signature for Gene Ontology (GO) and biological pathways. The lines represent protein-protein interactions mapped by STRING. The blue nodes are genes connected to TGF-β signaling, the red nodes represent genes involved in the regulation of the TGF-β production, yellow nodes represent genes involved in TGF-β receptors and green nodes represent the signaling by TGF-β members. Analysis was performed using R software with DESq2 package and the STRING webtool.

To gain biological insight into the functional role of the proposed gene set signature based on TCGA-HNSCC patients, an Ingenuity Pathway Analysis (IPA) and STRING enrichment analysis were performed. The IPA analysis revealed a significant enrichment of DEGs involved in cellular development, cellular growth and proliferation, cellular movement, cellular function, and cell-to-cell signaling (Table 2 and Supplementary Table S3). TGF-β signaling was the top ranked pathway in the analysis and five main genes (INHBA, SERPINE1, THBS1, ITGB6 and LTBP1) were highlighted as potential targets for further investigation (Figure 3A). These genes were found to be highly expressed in the group SOX2^Low^SOX9^High^ of the TCGA-HNSCC cohort (Supplementary Table S2). Furthermore, RNAseq data from HNO223 tumor cell lines knocked down for SOX2 or SOX9 demonstrated that 11 genes present in clinical samples were also present in clones knocked down for SOX2 or SOX9 (Figure 3B,C and Table 3). Four out of five of the predicted top candidate genes were also found dysregulated in either shSOX2 or shSOX9 cells, emphasizing once more the potential role of the TGF-β pathway.

### 3.4. SOX2 and SOX9 Are Independent in Gene Expression

**Figure 4:**
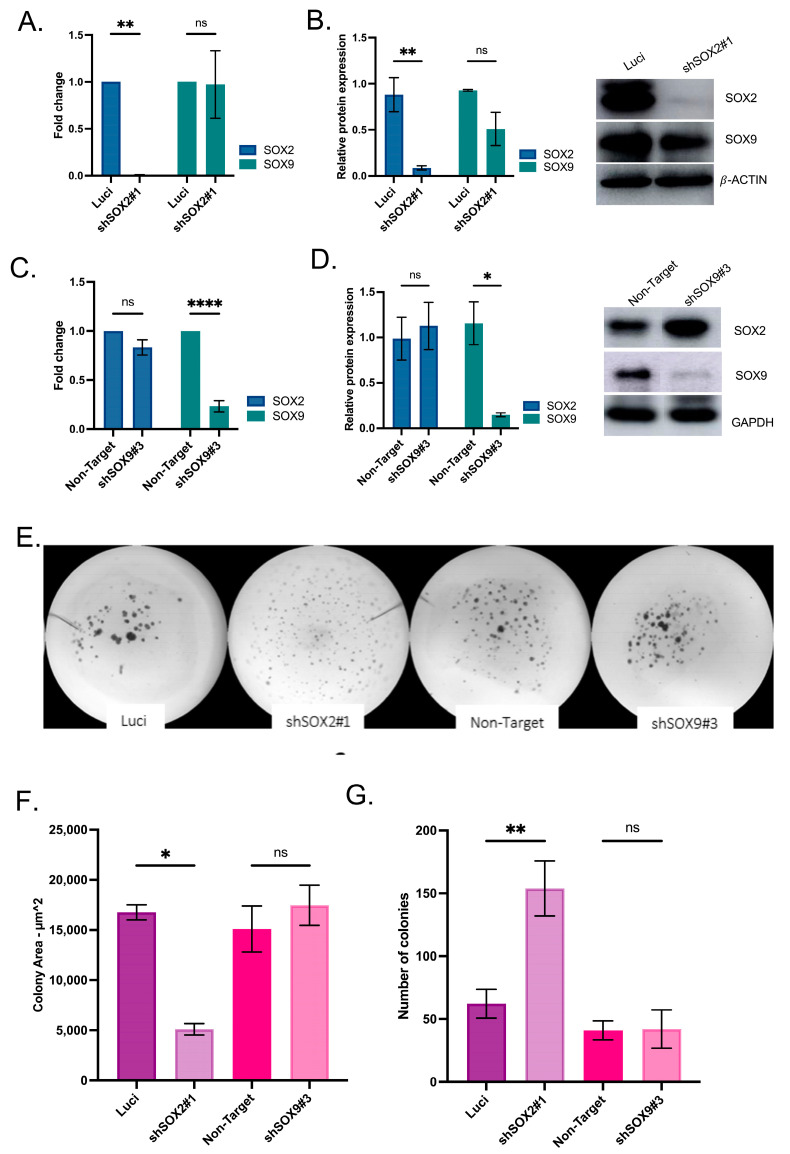
SOX2 and SOX9 present independent expressions in head and neck cell lines. (**A**). qRT-PCR for SOX2 and SOX9 mRNA expression in HNO223 shSOX2 cell lines. (**B**). Representative Western Blotting analysis of SOX2 and SOX9 protein expression after SOX2 silencing in HNO223 cell line. The uncropped blots are shown in Supplementary Material Figure S2. (**C**). qRT-PCR for SOX2 and SOX9 mRNA expression in HNO223 shSOX9 cell lines. (**D**). Western Blotting analysis of SOX2 and SOX9 protein expression after SOX9 silencing in HNO223 cell line. The silencing of SOX2 and SOX9 was confirmed and does not impact protein expression of individual genes. The uncropped blots are shown in Supplementary Material Figure S2. (**E**). Representative bright filed images of HNO223 control Luci, shSOX2#1, control non-target and shSOX9#3 in 3D Matrigel assay. (**F**). Spheres areas are measured after 10 days of cell seeding in a density equal to 1.5 × 10^3^ in Matrigel. HNO223 shSOX2 cells demonstrated smaller area as compared to shSOX9 and control cell lines. (**G**). Number of colonies were counted for colonies with minimum 50 μm of diameter. The shSOX2 cells presented higher number of colonies than shSOX9 and control cell lines. The shSOX2 cell line presented a higher tumor-initiation capacity as compared to shSOX9 cell line and controls. The relative protein expression for the genes of interest was calculated after normalization for the expression of GAPDH. The relative gene expression of the genes of interest was calculated after normalization for the expression of housekeeping genes (GAPDH and ACTB) using the 2^−ΔΔCT^ method. Two-Ways ANOVA in GraphPad Prism 9.0, *p* value was considered significantly, when lower than 0.05 and the bars represent SEM. ns, not significant; * *p* < 0.05, ** *p* < 0.01 and **** *p* < 0.0001.

To test whether the SOX2 and SOX9 expression have a direct impact on their expression, an existing SOX2 knockdown model [18] was used and a new SOX9 knockdown model was generated. Independent expression of SOX2 and SOX9 were observed, where the knockdown of SOX2 did not affect SOX9 at mRNA and protein expression levels (Figure 4A,B). The new SOX9 model with confirmed SOX9 silencing revealed no impact in SOX2 expression (Figure 4C,D). In addition, the potential to tumor sphere formation after SOX2 or SOX9silencing was tested. SOX2 silencing significantly increased the number of tumor spheres with a significant reduction in colony size when compared to SOX9 silencing and control cell lines (Figure 4E–G), whereas SOX9 silencing did not affect the tumor sphere formation or size of the colonies.

### 3.5. Radiation Impacts SOX2 and SOX9 Expression in Head and Neck Cell Lines

**Figure 5:**
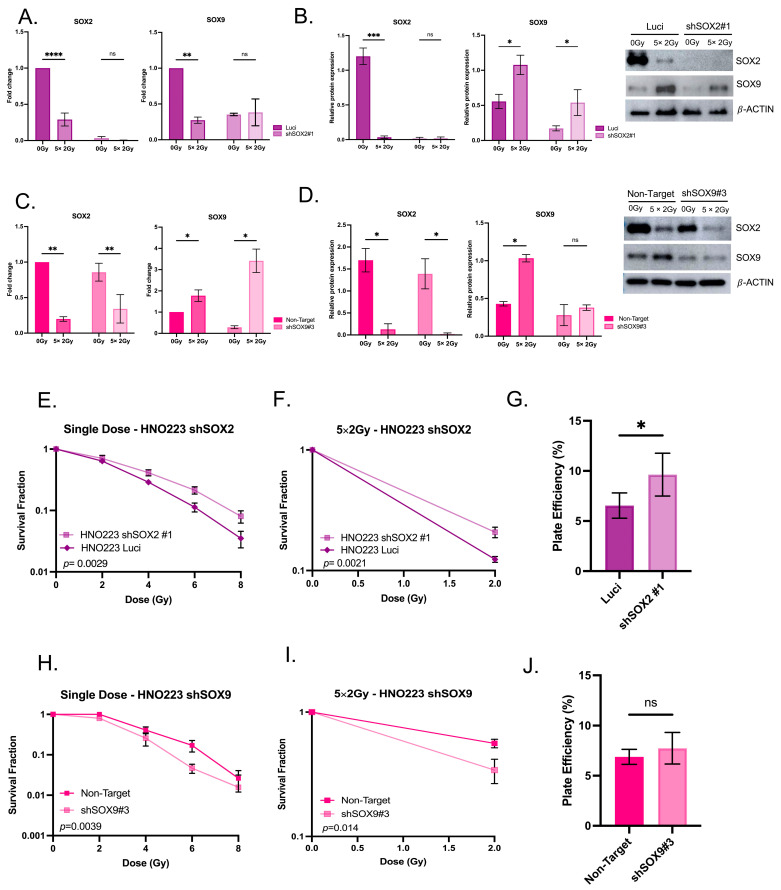
SOX2 knockdown promotes a radioresistant phenotype. (**A**). qRT-PCR for SOX2 (left) and SOX9 (right) mRNA expression in HNO223 shSOX2 cell lines after a fractionated irradiation protocol. (**B**). WB analysis for SOX2 (left) and SOX9 (right) protein in HNO223 shSOX2 cell lines after a fractionated irradiation protocol. The uncropped blots are shown in Supplementary Material Figure S2. (**C**). qRT-PCR for SOX2 (left) and SOX9 (right) mRNA expression in HNO223 shSOX9 cell lines after a fractionated irradiation protocol. (**D**). WB analysis for SOX2 (left) and SOX9 (right) protein in HNO223 shSOX9 cell lines after a fractionated irradiation protocol. Radiation promotes loss of SOX2 mRNA and protein expression in all tested cell lines. The uncropped blots are shown in Supplementary Material Figure S2. (**E**). 3D assay for a single dose in HNO223 shSOX2 cell lines. (**F**). 3D assay for a fractionated dose of 2 Gy daily for a 5-day period followed by a recovery time of 5 days in HNO223 shSOX2 cell lines. Spheres areas are measured after 10 days of cell seeding in a density equal to 1.5 × 10^3^. (**G**). Plate efficiency of HNO223 shSOX2 cell lines. Knockdown cells for SOX2 expression presented a radioresistant phenotype and a higher plate efficiency as compared with control cell lines. (**H**). 3D assay for a single dose in HNO223 shSOX9 cell lines. (**I**). 3D assay for a fractionated dose of 2 Gy daily for a 5-day period followed by a recovery time of 5 days in HNO223 shSOX9 cell lines. Spheres areas are measured after 10 days of cell seeding in a density equal to 1.5 × 10^3^. (**J**). Plate efficiency of HNO223 shSOX9 cell lines. Knockdown cells for SOX9 expression presented a radiosensitive phenotype as compared with control cell lines. SOX9 knockdown did not interfere with the plate efficiency. Linear Quadratic Model was used to calculate the radiation impact. The relative gene expression of the genes of interest was calculated after normalization for the expression of housekeeping genes (GAPDH and ACTB) using the 2^−ΔΔCT^ method. The relative protein expression for the genes of interest was calculated after normalization for the expression of Beta Actin. The appropriate statistical test was used in GraphPad Prism 9.0, *p* value was considered significantly, when lower than 0.05 and the bars represent SEM. ns, not significant; * *p* < 0.05, ** *p* < 0.01, *** *p* < 0.001 and **** *p* < 0.0001.

To investigate the effect of radiation in SOX2 and SOX9 expressions, we used a SOX2 knockdown (shSOX2) and a SOX9 knockdown (shSOX9) model. Head and neck cell lines were irradiated with a single dose of 2 Gy, 4 Gy, 6 Gy and 8 Gy or with a protocol of fractionated daily dose of 2 Gy for 5 days. Cell survival, tumor sphere formation and the SOX2 and SOX9 expression were analyzed in the cells silenced for SOX2 or SOX9 and compared to control cell lines (Figure 5E–J). Clonogenic assay showed that cells silenced for SOX2 exhibited a radioresistant phenotype in comparison to control cell lines (Figure 5E,F), whereas SOX9-silenced cells exhibited sensitivity to radiation in comparison to control cell lines (Figure 5H,I). Radiation promoted a drastic reduction of the expression of SOX2 at mRNA and protein levels (Figure 5A–D), when measured five days after the last dose. SOX9 was found to be upregulated on transcript as well as protein level after 5 × 2 Gy irradiation in shSOX2 and control cells (Figure 5B,D). In addition, 3D colonies derived from shSOX2 cells were smaller than shSOX9 and control cells, but had a higher number of spheres formed (Figure 4F,G). The cell lines silenced for SOX2 presented a higher plate efficiency as compared with other cells lines (Figure 5G,J). Taken together, these results suggest that tumor cells under irradiation downregulate their SOX2 expression while upregulating their SOX9 expression, and this inverse regulatory tendency may phenotypically increase their resistance to radiation.

### 3.6. Radiation Impacts Genes in the TGF-β Signaling Pathway

**Figure 6:**
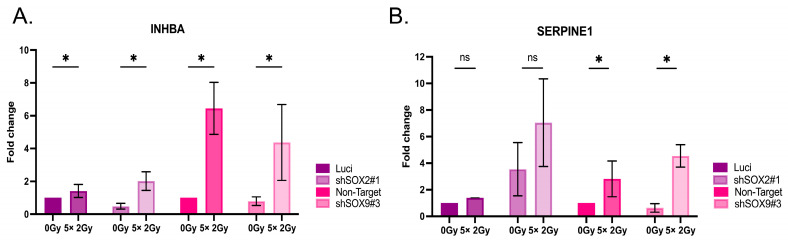
Radiation increases genes related to the TGF-β pathway. (**A**). qRT-PCR for INHBA mRNA expression in HNO223 shSOX2 and shSOX9 cell lines after a fractionated irradiation protocol. (**B**). qRT-PCR for SERPINE1 mRNA expression in HNO223 shSOX2 and shSOX9 cell lines after a fractionated irradiation protocol. Radiation promotes increased INHBA mRNA expression in all tested cell lines, while SERPINE1 showed an increase in silenced SOX9 cell lines. The relative gene expression of the genes of interest was calculated after normalization for the expression of housekeeping genes (GAPDH and ACTB) using the 2^−ΔΔCT^ method. Two-Ways ANOVA in GraphPad Prism 9.0, *p* value was considered significantly, when lower than 0,05 and the bars represent SEM. ns, not significant; * *p* < 0.05.

To investigate, whether ionizing irradiation affects top candidate genes in the above predicted gene signature derived of the inverse SOX2 and SOX9 expression in resistant and repopulating cancer cells, the transcript and protein levels in HNO223 cells subjected to the fractionated irradiation protocol (2 Gy/day for 5 days was investigated. qRT-PCR revealed an irradiation-induced significant increase in INHBA expression for all tested cell lines (Figure 6A) and SERPINE1 expression was significantly induced in the irradiated shSOX9 cell lines (Figure 6B).

### 3.7. SOX2 and SOX9 Present Difference Expression in Tumor Compartments

**Figure 7:**
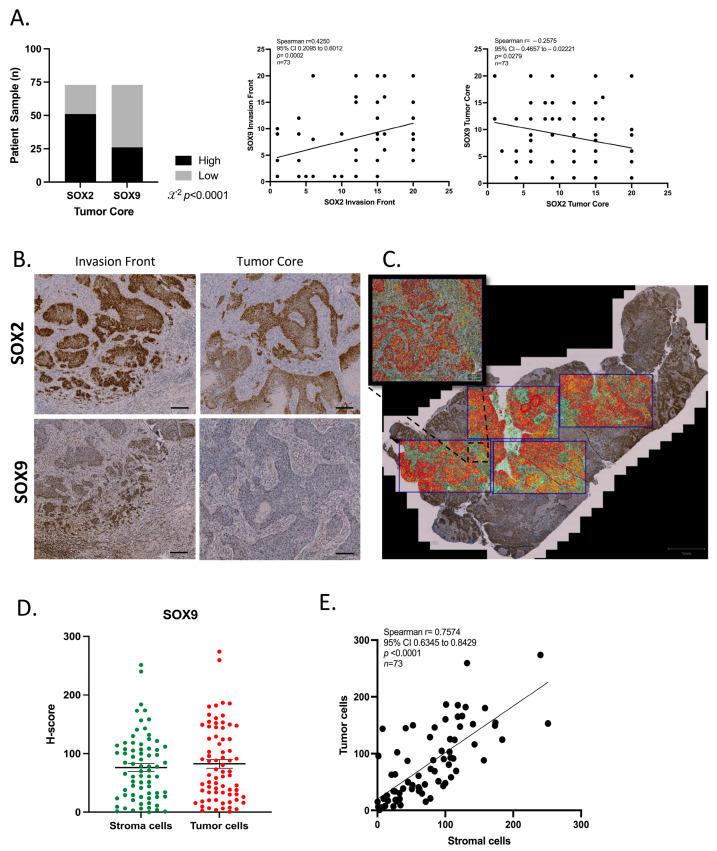
Characterization of SOX2 and SOX9 Protein Expression in Tumor Sections of the HIPO-HNC Cohort. (**A**). Left: Protein expression patterns in tumor core of the samples. Middle: Spearman correlation test according to the IRS for SOX2 and SOX9 at the tumor core. Right: Spearman correlation test according to the IRS for SOX2 and SOX9 in the invasion front of the samples. (**B**,**C**). Representative pictures of SOX2 and SOX9 expression at the invasion front and the tumor core. Analysis was performed in GraphPad Prism 9.0, *p* value was considered statistical significantly lower than 0.05. (**C**). Illustration of the representative areas of analysis (black rectangles) and annotations for stroma (green areas) and/or tumor cells (red areas) using QuPath image software (version 0.3.2). (**D**). Do plot shows H-scores for stromal and tumor cells in HNSCC samples. (**E**). Spearman correlation between H-scores for SOX9 in tumor cells and stromal cells. Plots and statistical analysis were prepared in Prism 9.0. *p* value lower than 0.05 was considered statistical. Scale bar = 100 μm.

To further explore the expression of SOX2 and SOX9 in cancer cells at protein level, immunohistochemical (IHC) staining was performed on FFPE tumor sections from the HIPO-HNC cohort [27]. Differences in the spatial expression pattern between SOX2 and SOX9 at protein levels were assessed in two distinct areas of the tumor tissue: the invasion front and the tumor core. For the semi-quantitative analysis of protein expression two parameters were defined: first, (I) five degrees of percentage for positive nuclei (1: negative staining, 2: 0–25%, 3: 26–50%, 4: 51–75% and 5: 76–100%), and second (II) three degrees of staining intensity (1: mild, 2: moderate and 3: strong) for each area. To additionally assess the relationship between SOX2 and SOX9 protein abundance and the microenvironment, a final immunoreactivity score (IRS) was calculated as described in [18]. A modest positive correlation was found for the IRS of both TFs at the invasion front (Spearman r = 0.4250, *p* = 0.0002) (Figure 7A,B), while a modest inverse correlation was evident at the tumor core (Spearman r= −0.2575, *p* = 0.0279) (Figure 7A,B). At the invasion front, a strong co-expression of both markers was observed (Figure 7B). SOX9 protein expression was higher in the invasive front compared to very few SOX9 positive cells in the tumor core. SOX2 staining was observed in both the tumor core and the invasive front. SOX2 protein expression was found exclusively in the tumor cell nuclei, whereas SOX9 was expressed not only in the tumor cells but also in some parts of the tumor stroma (Figure 7A,B). Taken together, these results showed a difference in spatial distribution of both TFs.

### 3.8. SOX9 Is Expressed in the Tumor Microenvironment

As a substantial expression of SOX9 positive cells was observed in the tumor microenvironment, the percentage of SOX9 positive cells and staining intensity were quantified using the QuPath software. Due to the differences in size of tumor samples, four areas of equal size per tumor were selected and were evaluated for SOX9 protein staining. Annotations for stromal and tumor areas, were individually made for selected areas of each respective sample. The total percentage of positive cells and the intensity of staining in each cell were then calculated, resulting in one H-score based on a median of 25.000 cells per sample (Figure 7C). Comparing SOX9 expression between stromal cells and tumor cells a similar H-score for both compartments in tumor samples were revealed (Figure 7D). Moreover, Spearman correlation analysis demonstrated a positive and significant correlation for the H-score between stromal cells and tumor cells (Figure 7E). In summary, a significant number of stromal cells in the TME were SOX9 positive, with intensity and proportion similar to tumor cells.

## 4. Discussion

### 4.1. Inverse SOX2 and SOX9 Expression Correlates with Disease Specific Survival in HNSCC

HNSCC is the most common malignance in the mucosa of the oral cavity, pharynx and larynx, characterized by a poor prognosis and a high aggressiveness, compromising facial structures and salivary glands functions by the heavy multimodal treatment [3,4]. There is a need to explore strategies to better stratify patients for therapies. SOX2 and SOX9 are both transcription factors that play important roles in embryonic development and tissue homeostasis [21,34]. The role of SOX2 in HNSCC is controversial, while it appears to have an oncogenic role in the formation of the tumor; its expression is likely modulated during the tumor evolution [34,35]. In our previous work, Bayo&Jou et al., we showed that patients with the worst response to therapy have low SOX2 expression and that after SOX2 silencing, HNSCC cells acquire a mesenchymal-like phenotype and migrate more compared to control cells [18]. SOX9 role in cancer is complex and context dependent. In some cancers, including HNSCC, SOX9 has been reported to exhibit both tumor-promoting and tumor-suppressive activities [36]. SOX9 overexpression has been associated with poor prognosis in some studies, while others have suggested a favorable prognostic role [20,37,38,39].

While most studies in HNSCC have a focus on the individual expression patterns of these genes, in this study we investigated the inverse SOX2 and SOX9 by establishing an integrative mathematical model to define patient stratification. We observed that the inverse relationship of both TFs correlates with disease specific survival of HNSCC patients. HNSCC patients with HPV negative status, treated with radiotherapy presenting SOX2^Low^SOX9^High^ expression had a reduced survival probability when compared to the group with SOX2^High^SOX9^Low^ expression. The prognosis and survival outcomes in HNSCC patients are determined by a combination of various molecular markers and clinical parameters. Therefore, the impact of SOX2 and SOX9 expression, including their inverse relationship, on DSS in HNSCC patients requires further investigation through clinical trials.

### 4.2. Gene Signature Based on the Inverse Expression of SOX2 and SOX9

Recently, a review of cancer hallmarks highlighted the importance of cellular plasticity. As a result of this plasticity, cancer cells can reprogram their gene expression in response to tumor development and therapeutic resistance [40]. Indeed, multiple studies have suggested an inverse correlation between the expression of SOX2 and SOX9 in cancer in a context dependent manner [25,41,42]. Here, in IHC staining analysis of the HIPO-HNC cohort we observed that gain or loss of expression depends on the region of the tumor. Sharma et al., demonstrated that a drug-induced adaptation was acquired upon loss of SOX2 with a concomitant gain in SOX9 in tumor cells [43]. Khorani et al., using DEGs gene related to SOX2 and SOX9, established an in silico prognostic risk model which suggests that inverse expression of both TFs may modulate cellular plasticity [41]. Thus, it may be that the tumor cells need a certain balance of expression to be able to migrate and/or resist treatment, suggesting a possible regulatory relationship between these two genes in HNSCC.

The interaction between these genes and other molecular pathways involved in HNSCC progression is complex and requires multifactorial investigation. For these reasons we explored a toolbox of computational analysis using SOX2 and SOX9 as potential biomarkers in HNSCC. Here we use mathematical modeling to define an integrative cutoff point for gene expression where the inverse SOX2 and SOX9 expression pattern might help in defining the clinical prognosis of HNSCC patients. We observed that clinical factors, such as the HPV status, subsite or radiation therapy, also play important roles.

Changes in gene expression might reflect mutual interactions between cancer cells and stromal cells of the TME, causing alterations in cellular plasticity, metastasis, and resistance to treatment [26,43,44]. A main hypothesis of this study was that the inverse regulation of these TFs might cause and/or reflect the consequences of altered gene expression, and thereby regulate tissue/cell morphology. Therefore, we strengthen our hypothesis by proposing a gene set signature based on the inverse expression of SOX2 and SOX9 for HNSCC patients with HPV negative tumors and treated with radiation. As a result, we could identify a set of 69 genes where the TGF-β signaling was predicted as a top up-regulated pathway. In addition, GSVA analysis demonstrated that patients with high scores for the proposed gene set signature presented a worse clinical outcome.

INHBA, SERPINE1 and THSB1 genes were the most highly expressed in the gene set signature and directly correlated with a group of patients with a SOX2^Low^SOX9^High^ that show a worse outcome in disease specific survival. Analyzing the overlap of our gene set with the one published in Khorani et al., we find INHBA and DUSP6 as predicted genes related to low SOX2 and high SOX9 indicating prominent activation of KRAS signaling [41]. Likewise, SERPINE1 also appeared in the overlap, corroborating with our indication of a possible role of TGF-β signaling in low SOX2 and high SOX9 patients. Shivaprasad et al., proposed that INHBA and SERPINE1 were top genes to stratify HNSCC patients HPV negative and postoperative radio(chemo)therapy [45].

In addition, INHBA is a gene that encodes proteins related to the TGF-β superfamily and high expression has been associated with invasive behavior of OSCC cells leading to a poor prognosis [46,47]. SERPINE1 encodes a member of the serine protease inhibitor superfamily, and it is associated with worse prognosis and radioresistance in HNSCC [45,48]. Lee et al., demonstrated that inhibition of SERPINE1 suppressed the self-renewal properties and increased radioresistance of HNSCC cells through downregulation of SOX2 [49]. THBS1 is a tumor-specific ECM protein, which is expressed mainly in the tumor microenvironment by stromal cells. THSB1 is stimulated by TGF-β signaling which initiates a cascade of activation through an integrin signaling network facilitating OSCC invasion [50].

Taken together, omics analyzes presented in this study support the hypothesis that the inverse SOX2/SOX9 expression is involved in tumor progression and resistance to treatment. The latter issue is of particular importance for HPV-negative HNSCC presenting SOX2^Low^SOX9^High^ expression, which are treated with radiotherapy and might have a better response to a different treatment strategy. The newly identified gene set signature presents a potential tool in patient stratification and advantage in treatment decisions.

### 4.3. TGF-β Signaling as One of the Top Canonical Pathways Regulated by Inverse SOX2/SOX9 Expression

The TGF signaling is a key process in tumorigenesis and regulates mechanisms either to suppress or promote tumor growth depending on the context [51,52]. INHBA, SERPINE1 and THSB1 genes were part of the gene set signature and highly expressed in the group of patients with a SOX2^Low^SOX9^High^ phenotype. Here, we demonstrated that SOX9 protein levels and INHBA expression are upregulated after radiation therapy, while SOX2 is downregulated. It is important to note that secretion of soluble molecules in the TME may activate or repress many cascades in the adjacent tumor tissue and is one way to regulate cancer cell profile [53]. In HNSCC, expression of activin A (INHBA) increases cellular migration, invasion and is related to unfavorable clinical prognosis [54]. Zhang et al., have shown that TGF-β secreted by tumor associated macrophages induces expression of SOX9 leading to an EMT phenotype in lung cancer cells, which was linked to tumor proliferation, migration and invasion [55]. Another study with lung cancer cells, demonstrated the involvement of TGF-β signaling in downregulation of SOX2 inducing EMT and promoting a change in cell morphology accomplished by a resistance to treatment [17].

In HNSCC, expression of INHBA increases cellular migration, invasion and unfavorable clinical prognosis [54]. Loomans et al., demonstrated that loss of epithelial Activin receptor type IB (ACVRIB) increases aggressiveness in SCC, which correlates inversely with high stromal expression of INHBA suggesting that the receptor modulates the tumor suppression activity of this protein [56]. Tsai et al., reported that EGFR is activated by INHBA in OSCC cells via the non-canonical PI3K/SPI pathway, and high expression of EGFR was significantly associated with poor clinical prognostic [57]. In a recent study, a risk model based in inverse SOX2/SOX9 expression also identified EGFR signaling as a candidate for HNSCC patients with SOX2 low and SOX9 high expression pattern [41]. Here, we demonstrated that INHBA expression is highly expressed after a fractionated dose of radiation in all tumor cells, while SERPINE1 expression was highly increased in the cells with knockdown of SOX9 after irradiation. It was also observed that SERPINE1 was downregulated after SOX9 knockdown, and that radiation induced upregulation. The findings suggest a possible link between these two genes that warrants further study. The functional importance of this inverse association might be linked to the proposed gene set signature, which is either the cause or the consequence of the interactions with stromal cells of the tumor environment via TGF-β signaling. The observation that HNSCC patients with low SOX2 and high SOX9 expression, identified here as a group with low DSS, highly expressed five key genes (INHBA, SERPINE1, THBS1, ITGB6 and LTBP1) of the TGF-β signaling pathway suggests a possible mechanistic explanation for the role of SOX2 and SOX9 in HNSCC patients’ survival.

### 4.4. SOX9 in the Tumor Microenvironment

The tumor microenvironment is composed of cancer associated fibroblasts (CAFs), blood and lymph vessels, immune cells, growth factors, cytokines and they are in constant interplay with cancer cells [55,58,59,60]. Here we observed that cancer cells at the invasive front presented a positive staining for both TFs, though the staining intensity differed from individual cancer cells. This was particularly evident for cancer cells at the border of the invasive areas, which presented higher expression for SOX9 but lower SOX2 expression. Altogether, the results presented here demonstrate that a prominent SOX2 expression was only detected in cancer cells, while SOX9 expression was also detected in stromal cells of the TME.

Spatial and temporal differences in SOX9 expression could be explained by distinct modes of regulation, including epigenetic modification, differences in the TME, and response to treatments [61,62,63,64]. Here, we demonstrated that SOX9 plays an important role during tumor progression being highly expressed in cancer cells at the invasive front, but lower expression has been detected in cancer cells of the tumor core. In addition, the intensity of the expression in the stroma cells was positively correlated with the intensity of expression by tumor cells. In a recent study, the cytoplasmic SOX9 levels were identified as a prognostic biomarker in OSCC [65]. However, SOX9 cytoplasmic staining was neither observed at the tumor core nor at the invasive front of tumor samples of the HIPO-HNCC cohort, including OSCC.

These findings strengthen our claim that high SOX9 expression could serve as surrogate marker for a TGF-β-enriched TME, which might also explain the presence of SOX9-positive stromal cells in human tumor samples of the HIPO-HNC cohort. Haga et al., demonstrated that CAFs at the invasion front of OSCC up-regulate SOX9 expression through secretion of TGF-β1 which promotes migration and invasiveness of OSCC cells [66]. Riemenschnitter et al., demonstrated that high SOX9 expression in tumor stromal cells correlates with lower overall survival in breast cancer patients treated with neoadjuvant therapy [67].

The role of SOX9 expression in diverse stromal cells of the TME is not explored yet and these findings provide new insights into the potential role of SOX9 in the mutual crosstalk of cancer cells with the microenvironment. Moreover, IPA analysis showed numerous pathways related to cell adhesion, migration, and cellular motility, especially related to the candidate genes highly expressed in HNSCC patients belonging to SOX2^Low^SOX9^High^ group. These data not only suggest a possible role of the TME in the regulation of an inverse SOX2-SOX9 expression pattern in cancer cells, but also indicate the presence of a SOX9-related gene-regulatory network in stromal cells, which modulates characteristic trails of the TME. This spatial difference and the difference in intensity of cellular expression might explain the difficulty in elucidating the contribution of both TFs during metastasis and resistance to treatment in HNSCC, but also other solid tumors.

Additional future experiments should explore a larger panel of EMT markers and oncogenic pathways related to cancer cell dissemination in the newly established SOX9 knockdown model utilizing more complex in vitro as well in vivo models. Our results support the hypothesis that the inverse SOX2^Low^SOX9^High^ expression is associated with accelerated dissemination of HNSCC cells, which could contribute to tumor relapse and metastasis.

### 4.5. Cancer Stem Cells, the Tumor Microenvironment and Radiation Treatment

The tumor microenvironment plays an important role in cancer cell plasticity and regulation of the CSC state, and soluble factors secreted by stromal cells have the potential to dedifferentiate cancer cells into a CSC phenotype [68]. Here we show an irradiation-induced upregulation of SOX9 and INHBA, a soluble factor related to TGF-β signaling. These data are in line with other studies demonstrating an increase in INHBA expression upon irradiation [47,69,70]. Hence, candidate genes of newly identified gene set signature related to inverse SOX2/SOX9 expression has not only innovative potential to identify cancer cell intrinsic modes of radioresistance but could also potentially predict mechanisms of immune escape during radiotherapy. We speculate that SOX9 expression could be a downstream target of TGF-β signaling that might be released in the TME in response to an inflammatory process. However, more experimental evidence needs be explored to elucidate the axis TGF-β/SOX2/SOX9.

Radiotherapy is the first line of treatment in many tumor entities, including HNSCC and can be combined with other modalities to improve the clinical outcome of cancer patients [68]. A growing body of evidence supports the assumption that acquisition of CSC properties is associated with a radioresistant phenotype [32,34,71,72]. We demonstrated in the bioinformatic analysis that HPV-negative HNSCC patients with a SOX2^Low^SOX9^High^ expression pattern present a worse survival probability under radiotherapy. Chung et al., suggested that SOX2 enhances the effects of irradiation in HNSCC cell lines, and improves the prognosis of patients which might benefit from radiotherapy [73]. This assumption is further supported by our experimental data derived from in vitro cell cultures. First, the survival of OSCC cells upon irradiation was associated with relatively low SOX2 but high SOX9 expression. Second, the acquisition of a radioresistant phenotype was evident in SOX2 silenced OSCC cell line. Finally, fractionated irradiation revealed a further reduction in SOX2 levels, while SOX9 protein expression was increased. These data indicate a protective role of high SOX9 expression in cancer cells during radiotherapy, which is supported by Roche et al. [61]. They demonstrated in a mouse model an accelerated proliferation but reduced radioresistance of intestinal stem cells with a SOX9 knockout as compared to controls. Sharma et al., also proposed that stressor factors could increase SOX9 expression causing treatment resistance [43].

In addition, numerous studies have supported the assumption that SOX2 expression is associated with a tumor-initiation capacity [34,35,74]. However, Sharma et al., have demonstrated that in OSCC cells resistance to cisplatin does not depend on SOX2 levels or its tumor-initiation capacity, suggesting the involvement of another stem cell factor promoting the proliferation and growth of tumor cells [43]. Indeed, in a 3D Matrigel assay SOX2-silencing in a cancer cell line with concomitant SOX9 expression present a higher tumor-initiating capacity compared with control cells. These colonies presented a reduced size compared to controls which might be due to the altered migratory behavior of the SOX2-silenced cells. Interestingly, Garcia et al., reported that in colorectal adenocarcinoma cell lines, higher SOX9 expression was associated with CSC properties and metastasis. The cell lines with high SOX9 expression exhibited a higher number of tumor sphere formation as compared to controls [19].

It is likely that the impact of SOX2 and SOX9 on the response of cancer cells to irradiation is highly context dependent and critically depends on other epigenetic and genetic alterations. Accordingly, a more comprehensive analysis in future studies, including a larger set of cancer cell lines and adequate in vitro as well as in vivo pre-clinical models is needed to unravel the complexity and context dependent function of both TFs during irradiation.

## 5. Conclusions

SOX2 and SOX9 predicted a gene set signature modulating radiosensitivity, and TGF-b signaling appears to be one of the key pathways in promoting candidate genes overexpression. Furthermore, SOX2 expression is reduced under irradiation, while SOX9 is highly expressed. It is important to note that these results pave the way for further investigation of transcription factors’ potential use for predicting HNSCC patients’ outcomes. These newly uncovered evidence establishes a connection between SOX2/SOX9 gene regulation and TGF-β signaling and identifies the TME as an important factor in promoting treatment resistance in cancer cells expressing SOX2 and/or SOX9. To develop novel strategies for patient stratification and low-toxicity therapies, it is important to understand the molecular mechanisms underlying functional crosstalk between TGF-β signaling and SOX-related genes.

## Figures and Tables

**Table 1:** Histopathological and Clinical Data of the TCGA-HNSCC Cohort.

Feature		SOX2^High^SOX9^Low^ *n* (%)	SOX2^Low^SOX9^High^ *n* (%)	*p*
Patients (*n*)		260	214	
Vital Status	Alive	157 (60.4)	124 (57.9)	0.63
	Dead	103 (39.6)	90 (42.1)	
Overall Survival 5-year	0	167 (64.2)	132 (61.7)	0.56
	1	93 (35.7)	82 (38.3)	
Disease Specific Survival 5-years	0	195 (75)	155 (72.4)	0.14
	1	65 (25)	59 (27.6)	
HPV status ^1^	Negative	194 (77)	196 (98)	**0.0001**
	Positive	58 (23)	4 (2)	
Alcohol	No	77 (30.3)	68 (32.4)	0.68
	Yes	177 (69.7)	142 (67.6)	
Smoking	No	52 (20.4)	55 (26)	0.15
	Yes	203 (79.6)	156 (74)	
Gender	Female	55 (21.2)	67 (31.3)	**0.01**
	Male	205 (78.8)	147 (68.7)	
Age (median, range)		60 (19, 84)	61 (24, 90)	>0.99
Subsite	Hypopharynx	4 (1.5)	6 (2.8)	**0.0001**
	Larynx	76 (29.2)	29 (13.5)	
	Oral Cavity	122 (49.9)	169 (78.9)	
	Oropharynx	58 (19.4)	10 (4.8)	
Pathological Grading	G1	27 (10.4)	31 (14.5)	0.31
	G2	154 (59.3)	132 (61.7)	
	G3	67 (25.7)	47 (21.9)	
	G4	2 (0.7)	0	
	GX	8 (3)	4 (1.9)	
Tumor Size	cT1	19 (7.3)	14 (6.5)	0.74
	cT2	76 (29.2)	56 (26.2)	
	cT3	67 (32.6)	55 (25.7)	
	cT4	91 (35)	81 (37.8)	
	cTX	6 (2.3)	5 (2.3)	
Lymph Node metastasis	cN0	130 (50)	93 (43.4)	0.40
	cN1-3	119 (45.7)	110 (51.4)	
	cNX	10 (3.9)	8 (3.8)	
Distant metastasis	M0	247 (95)	198 (92.5)	0.47
	M1	1 (0.3)	3 (1.4)	
	MX	11 (4.2)	9 (4.2)	
Tumor Size	pT0	1 (0.4)	0	0.76
	pT1	23 (8.8)	20 (9.3)	
	pT2	73 (28)	53 (24.8)	
	pT3	49 (18.9)	40 (18.7)	
	pT4	85 (32.7)	78 (36.4)	
	pTX	20 (7.7)	12 (5.6)	
Lymph Node metastasis	pN0	90 (34.6)	71 (33.2)	0.52
	pN1	121 (46.6)	107 (50)	
	pNX	39 (15)	25 (11.7)	
pM	pM0	95 (36.5)	84 (39.2)	0.42
	pM1	1 (0.4)	0	
	pMX	36 (13.8)	24 (11.2)	
Pathological Staging	Stage I	14 (5.3)	11 (5.1)	0.67
	Stage II	38 (14.7)	28 (13.1)	
	Stage III	43 (16.5)	30 (14.1)	
	Stage IVA	121 (46.5)	116 (54.2)	
	Stage IVB	7 (2.7)	4 (1.8)	
Neoadjuvant Treatment	No	257 (98.8)	212 (99)	>0.99
	Yes	3 (1.2)	2 (1)	
Radiation	No	72 (32.2)	71 (36.8)	**0.01**
	Yes	152 (67.8)	122 (63.2)	
Additional Radiation Therapy	No	54 (58.7)	56 (70)	0.15
	Yes	38 (41.3)	24 (30)	

Chi-squared test (*χ*^2^) test, *p* values < 0.05 are indicated in bold. ^1^ HPV status—positive for viral DNA and transcripts. c: clinical evaluation, p: pathological evaluation. Missing data were excluded for the analysis.

**Table 2:** Molecular and Cellular Functions Affected by the Signature Gene Set.

Function Name	*p*-Value	Number of Molecules
Cellular Development	4.70 × 10^−2^–2.02 × 10^−6^	15
Cellular Growth and Proliferation	4.70 × 10^−2^–2.02 × 10^−6^	15
Cellular Movement	3.86 × 10^−2^–5.65 × 10^−4^	12
Cellular Function and Maintenance	3.16 × 10^−2^–9.01 × 10^−4^	4
Cell-To-Cell Signaling and Interaction	4.30 × 10^−2^–1.19 × 10^−3^	9

**Table 3:** Genes expressed in TCGA-HNSCC gene signature and SOX2 or SOX9 knockdown in head and neck cell lines.

	HNO223 shSOX2		HNO223 shSOX9	
Gene	log2FoldChange	padj	log2FoldChange	padj
INHBA	−1.5583044	0.00110584	−1.1062817	6.67 × 10^−5^
SERPINE1	3.09471999	4.07 × 10^−13^	−1.2736951	0.00033041
THBS1	−1.3113053	0.00875671	−2.3902272	0.00427583
LTBP1	4.60426832	3.43 × 10^−28^	0.91433761	0.03975225
GCHFR	−1.1650005	0.01166647	0.69411435	0.04855759
PRDM8	−1.4274595	0.00463216	0.39562131	1.38 × 10^−5^
DUSP6	−3.3943627	9.16 × 10^−10^	−1.8248729	5.81 × 10^−7^
TNS4	−9.1657433	4.84 × 10^−59^	1.07067784	0.00040597
TINAGL1	−5.9954209	8.60 × 10^−54^	−0.60474	0.04086991
LAMC2	−3.2760053	5.55 × 10^−16^	−0.9932665	5.14 × 10^−5^
SERPINB7	−3.9976105	1.03 × 10^−15^	−1.0997699	0.00022133
SOX2	−7.6332362	9.28 × 10^−5^	1.29689157	0.0589497
SOX9	−2.0600676	1.76 × 10^−6^	−1.9138004	4.31 × 10^−10^

## Data Availability

All data that support the findings of this study are included in the manuscript or in Supplementary Materials. The results shown here are in part based upon data generated by the TCGA Research Network (http://cancergenome.nih.gov accessed on 24 November 2021). RNA-seq count data of The Cancer Genome Atlas (TCGA) are openly available at https://portal.gdc.cancer.gov accessed on 25 November 2021. The Heidelberg Center for Personalized Oncology (HIPO) HNC data set are available in GEO database with access number GSE117973. Sequencing RNA data presented in this study are available on request from the corresponding author.

## Supplementary Materials

The following supporting information can be downloaded at: https://www.mdpi.com/article/10.3390/cancers16020439/s1, Figure S1: Characteristics of SOX2 and SOX9 Expression in HNSCC Patient Cohorts; Figure S2: Original Western Blot images cited in Figure 4B,D and Figure 5B,D; Table S1: Co-expressed genes associated with inverse SOX2 and SOX9 expression in TCGA-HNSCC cohort; Table S2: Gene set signature based on the inverse SOX2 and SOX9 expression; Table S3: Ingenuity Pathway Analysis—Top Canonical Pathways; Table S4: Primer qPCR.
