# Supplementary material for: The Role of SOX2 and SOX9 in Radioresistance and Tumor Recurrence
Source: Cancers (Basel). 2024 Jan 19;16(2):439. doi: 10.3390/cancers16020439 (PMC10814462; doi:10.3390/cancers16020439)
Supplement: Supplementary file 1 [file cancers-16-00439-s001.zip › cancers-2776517-supplementary/Supplementary Figure S2.pdf]

Article,

The Role of SOX2 and SOX9 in Radioresistance and Tumor Recurrence.

Original Images for Blots and Gels Requirements

Supporting Information files

Silvia Barbosa[1,3,6*], Natalia Laureano Koerich[1,2], Wahyu W. Hadiwikarta [1], Fernanda Visioli [2], Mahnaz Bonrouhi [1], Kinga Pajdzik [4], Christel Herold-Mende [3], Gustavo Eidt [1], Renan Langie[1,2], Marcelo Lazzaron Lamers [6], Fabian Stögbauer 10, 11 , Jochen Hess [4], Ina Kurth [1,8,9#] and Adriana Jou [5,7#].

Correspondence: Silvia Barbosa (slgentil@hotmail.com)

The preparation procedure for the images in this manuscript has been as follows:

1. Experimental Protocol:

First, a 12% acrylamide/bis-acrylamide gel was prepared. Equal amounts of samples (10μg) were loaded onto the gel. After electrophoresis, the separated proteins were transferred to a PVDF membrane (Millipore). The membranes were incubated overnight with primary antibodies against SOX2 (α-rabbit; 1:1000; Cell Signaling - D6D9) or SOX9 (α-rabbit; 1:1000; Cell Signaling - D8G8H). The secondary antibody, horseradish peroxidase (HRP; Cell Signaling - 7074S), was incubated for 1 hour at room temperature. The signal was then detected using chemiluminescence LumiGLO® solution (Cell Signaling - 7003S) on an ImageQuant LAS500 system (GE Healthcare Life Science). After primary antibody capture images, the membrane was washed with 0.1% PBS-T for 30 minutes, blocked for 30 minutes and the housekeeping gene was applied for one hour at room temperature, followed by secondary antibody incubation for 30 minutes. Finally, chemiluminescence LumiGLO® solution (Cell Signaling - 7003S) was used to detect the signal again using the ImageQuant LAS500 system (GE Healthcare Life Science). For both quantity and quality control of the protein lysates, the following housekeeping genes were used for loading: Beta-Actin (alpha-rabbit; 1:1000; Cell Signaling - D6A8) and/or GAPDH (14C10, alpha-rabbit; 1:1000; Cell Signaling).

2. Western blotting quantification steps:

2.1. Images have been uploaded to the ImageJ software;

2.2. The settings of the measurements are checked: Analyze -> Set Measurements -> ONLY the "Gray Mean Value" has been selected.

2.3. Images were loaded by drag&drop and adjusted to 32-bit color (Image -> Type -> 32-bit);

2.4. Selection as Region of Interest (ROI): Select the "Rectangle" tool from ImageJ. Draw a frame around the largest band of the row with the target protein following ladder sizes. Place the frame on the first band. Click on "Measure" in the "Analyze" menu and record the values in an Excel sheet. Open the ROI of the other band/rows or of the loading controls and make the measurements for the bands in the same way. The ROIs are kept the same size for all target rows.

2.5. Excel calculates each protein's value by ratio to the corresponding housekeeping gene in the same well.

All images present in this file are original images acquired using the ImageQuant LAS500 system (GE Healthcare Life Science). Images present in the main text have been cropped from indicated images and any rearrange in wells are indicated in the original image. Since this work was part of a larger project, not all membranes were used solely for the data presented. They also were used to validate other cell lines over the course of the data acquisition. The bands used for the protein analysis of the presented data are marked with a red rectangle around the cell line name and membranes are numerically labelled.

Figure 4. SOX2 and SOX9 present independent expressions in head and neck cell lines.

B. Western Blotting analysis of SOX2 and SOX9 protein expression after SOX2 silencing in HNO223 cell line**.**

For this analysis, the relative expression of SOX2 and SOX9 in HNO223 control (Luci) and silenced (shSOX2#1) cells was calculated using membranes **M1 to M6**. The letter *p* followed by a number indicates the passage used, the red rectangles indicate the clones used for analysis, and the arrow indicates the line corresponding to the predicted size band. Representative image in the main text were cropped from M3 and M4.

D. Western Blotting analysis of SOX2 and SOX9 protein expression after SOX9 silencing in HNO223 cell line.

For this analysis, the relative expression of SOX2 in HNO223 control (non-target) and silenced (shSOX9#3) cells was calculated using membranes **M16 to M18**. The letter *p* followed by a number indicates the passage used. The red rectangles indicate the clones used for analysis and the arrow indicates the line corresponding to the predicted size band. The rearrangement of the columns was done by cropping from the M18 by closing the lane to the target cell lines that are close to each other.

For this analysis, the relative expression of SOX9 in HNO223 control (Non-Target) and silenced (shSOX9#3) cells was calculated using membranes **M19 to M21**. The letter *p* followed by a number indicates the passage used. The red rectangles indicate the clones used for analysis and the arrow indicates the line corresponding to the predicted size band. The rearrangement of the columns was done by cropping from the M21 by closing the lane to the target cell lines that are close to each other.

Figure 5. Figure 5. SOX2 knockdown promotes a radioresistant phenotype.

B. WB analysis for SOX2 (left) and SOX9 (right) protein in HNO223 shSOX2 cell lines after a fractionated irradiation protocol**.**

For this analysis, the relative expression of SOX2 and SOX9 in HNO223 control (Luci) and silenced (shSOX2) cells was calculated using membranes **M7 to M10 and M13 and M14** for condition 0Gy (control non irradiated) and 5x2Gy (irradiated cells). The letter *p* followed by a number indicates the passage used, the red rectangles indicate the clones used for analysis, and the arrow indicates the line corresponding to the predicted size band.

D. WB analysis for SOX2 (left) and SOX9 (right) protein in HNO223 shSOX9 cell lines after a fractionated irradiation protocol.

For this analysis, the relative expression of SOX2 and SOX9 in HNO223 control (Non-Targert) and silenced (shSOX9) cells was calculated using membranes **M11 to M15** for condition 0Gy (control non irradiated) and 5x2Gy (irradiated cells). The letter *p* followed by a number indicates the passage used, the red rectangles indicate the clones used for analysis, and the arrow indicates the line corresponding to the predicted size band.

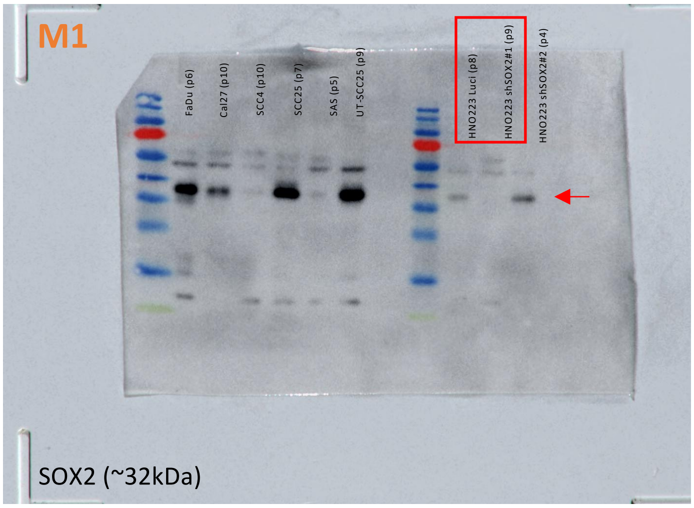

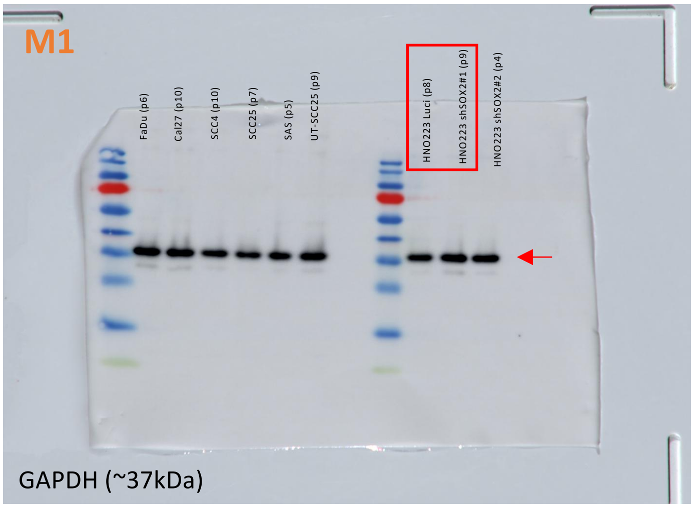

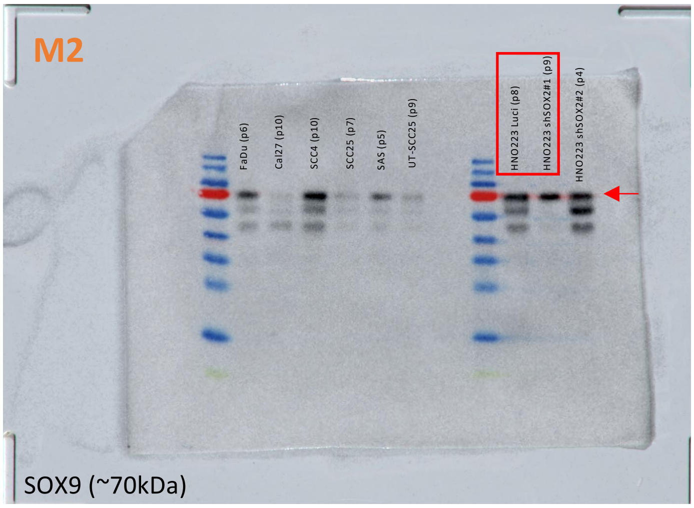

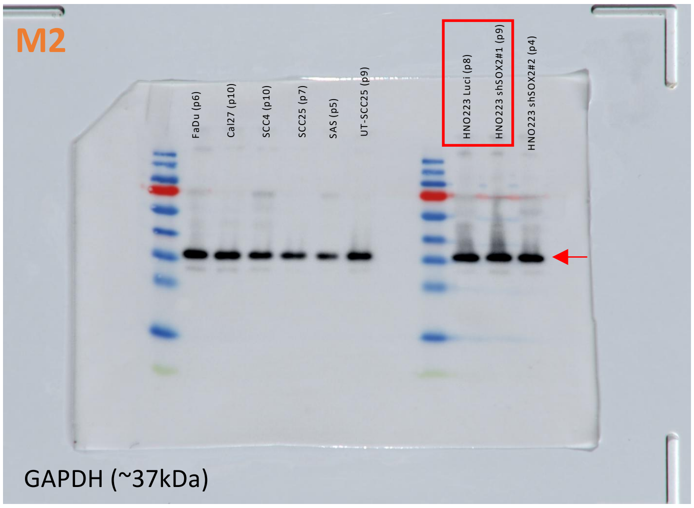

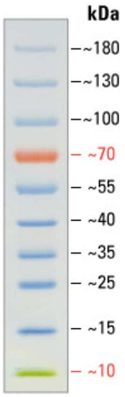

PageRuler™ Prestained Protein Ladder *(Thermo Fisher #26616)*

Representative image in main text.

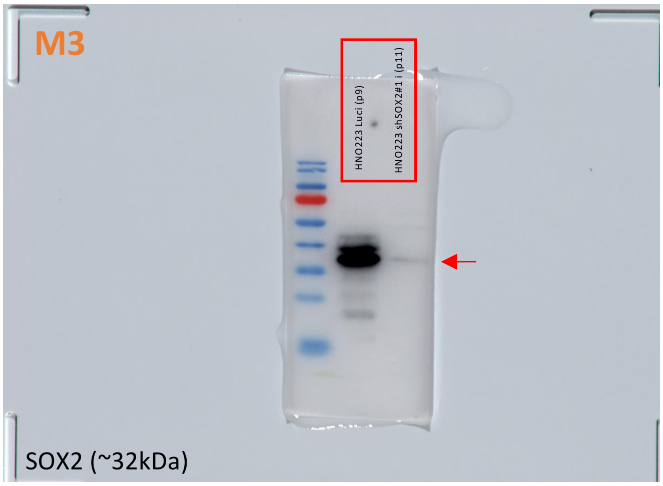

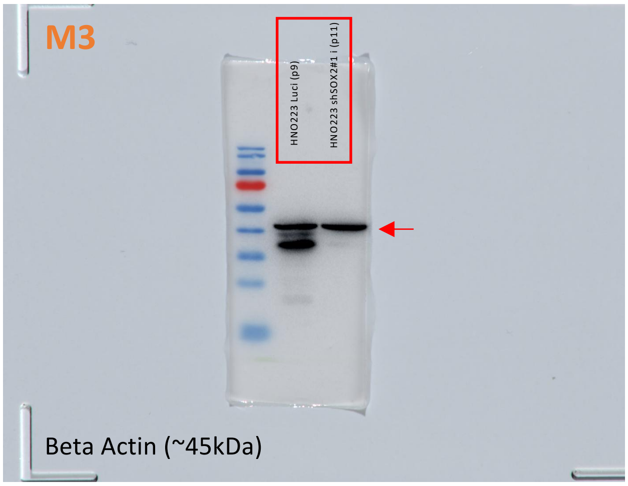

Representative image in main text.

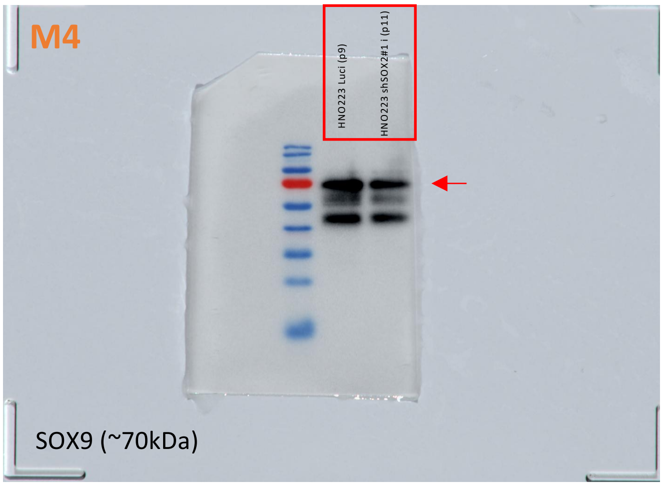

Representative image in main text.

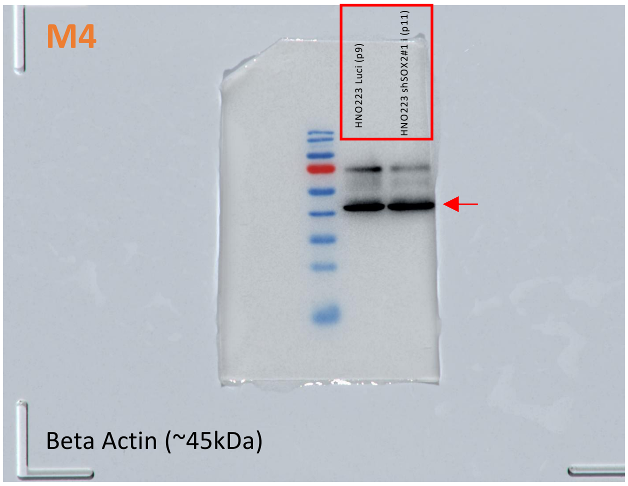

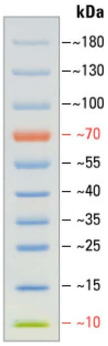

PageRulerTM Prestained Protein Ladder (Thermo Fisher #26616)

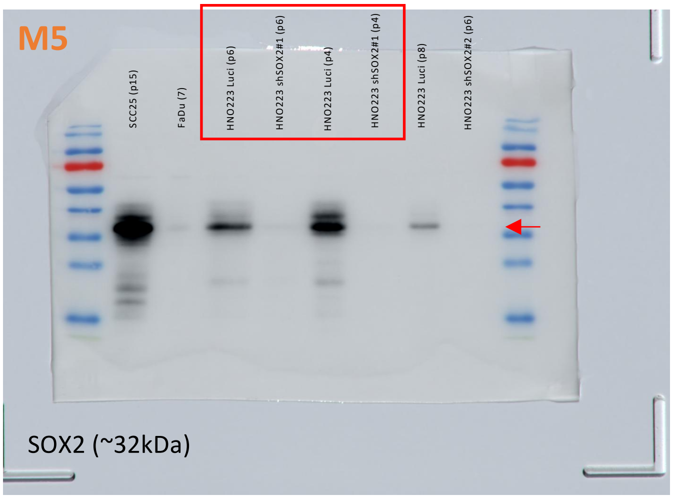

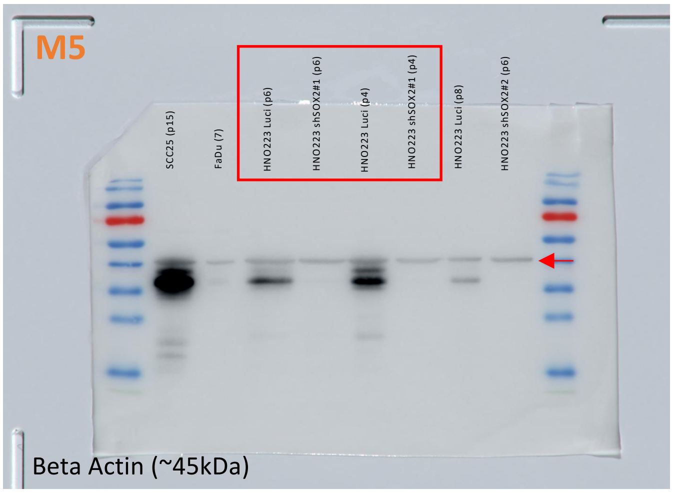

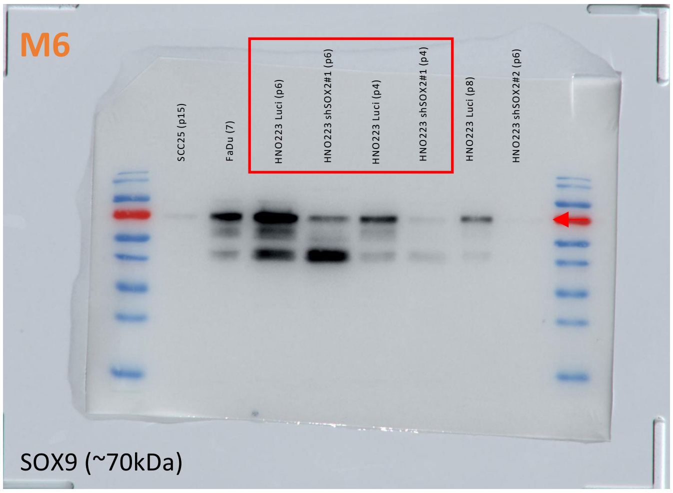

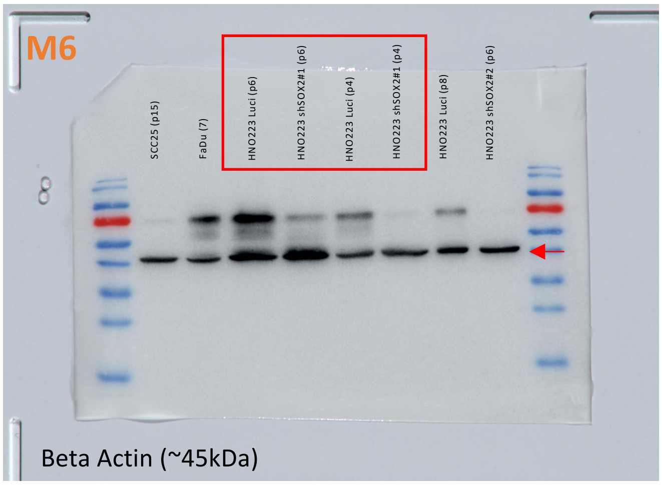

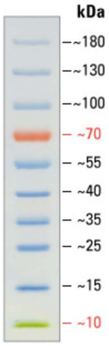

PageRulerTM Prestained Protein Ladder (Thermo Fisher #26616)

PageRulerTM Prestained Protein
Ladder (Thermo Fisher #26616)

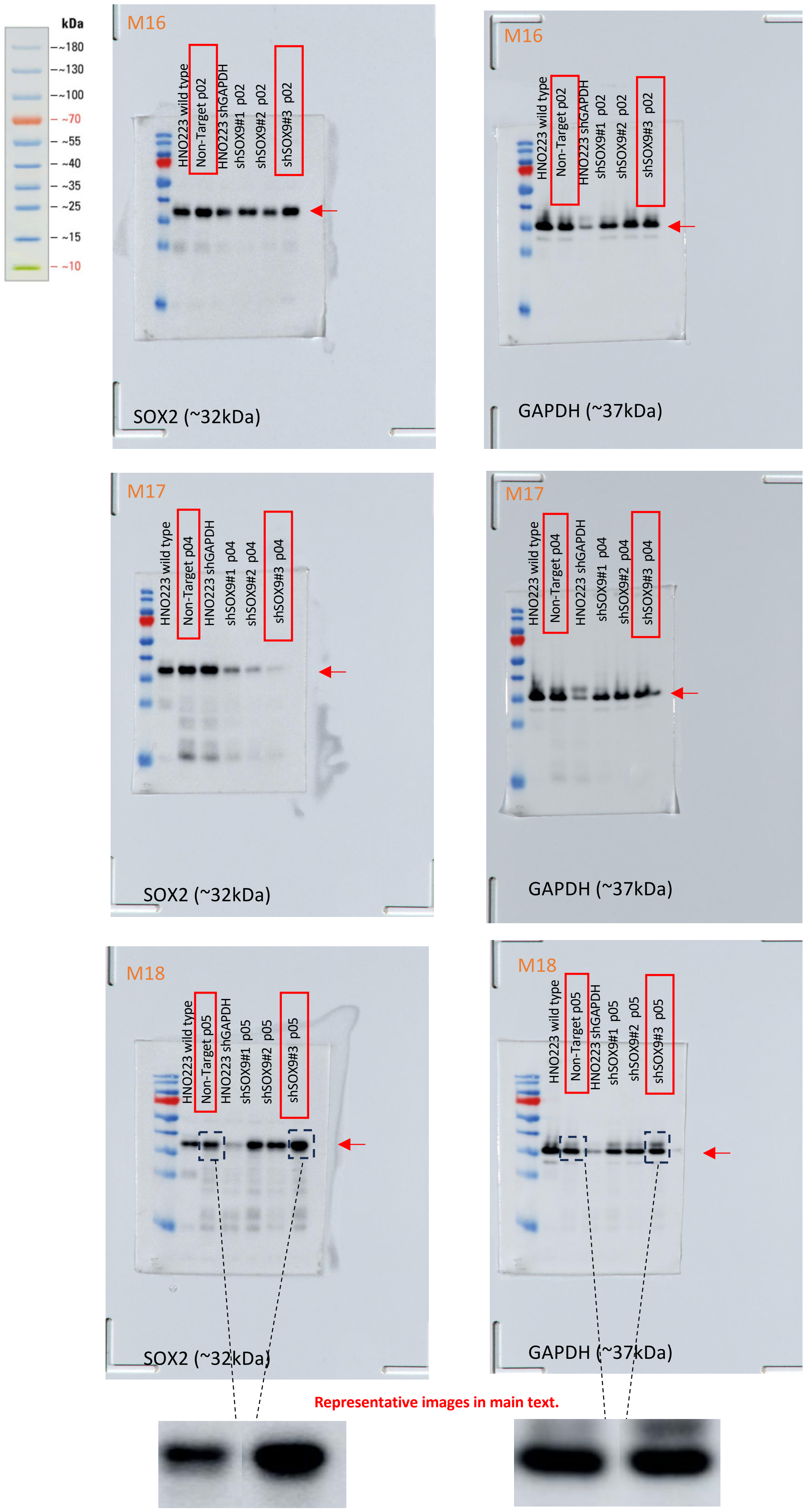

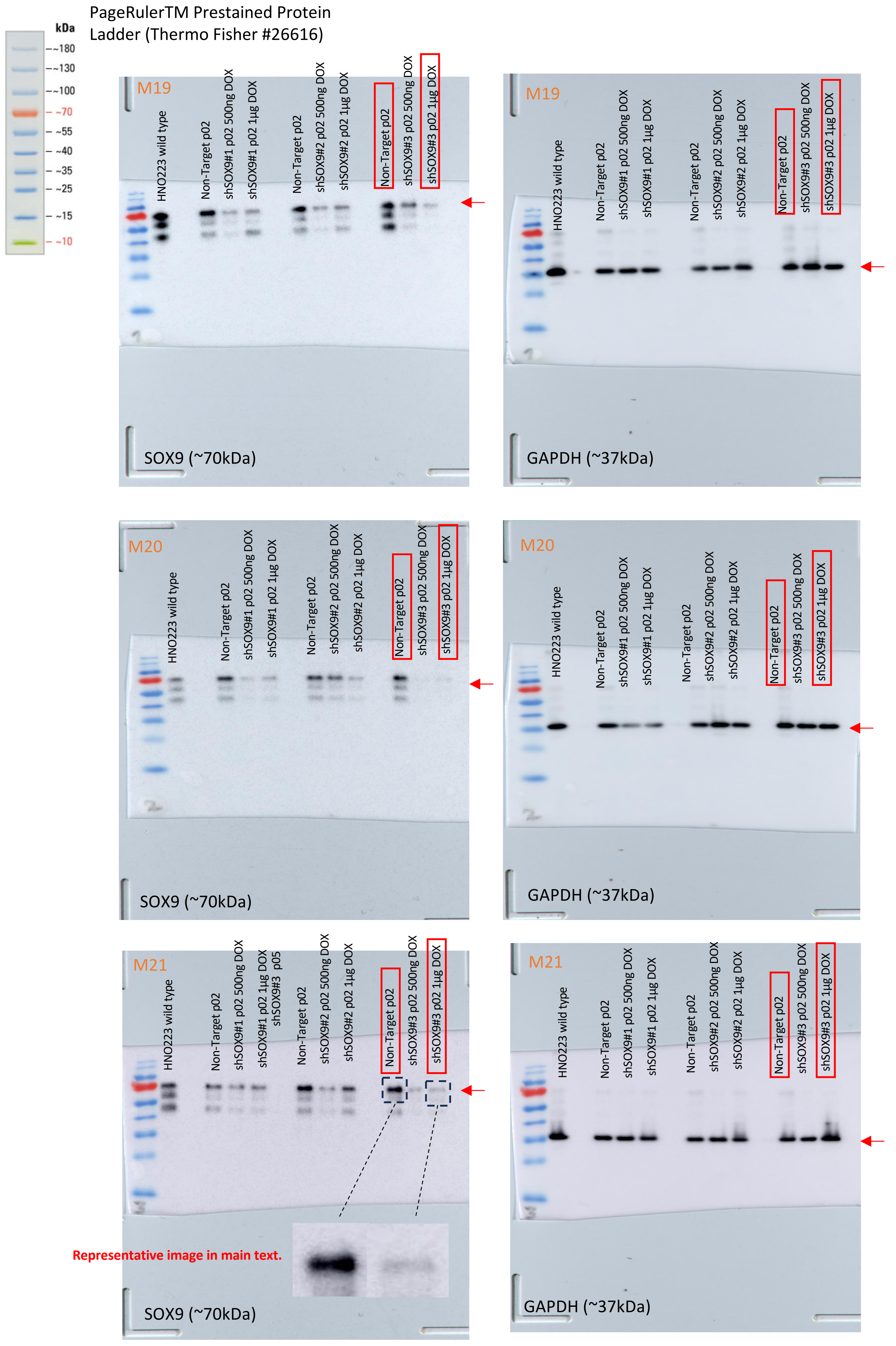
PageRulerTM Prestained Protein Ladder (Thermo Fisher #26616)
kDa
~180
~130
~100
~70
~55
~40
~35
~25
~15
~10
M19
HNO223 wild type
Non-Target p02
shSOX9#1 p02 500ng DOX
shSOX9#1 p02 1µg DOX
Non-Target p02
shSOX9#2 p02 500ng DOX
shSOX9#2 p02 1µg DOX
Non-Target p02
shSOX9#3 p02 500ng DOX
shSOX9#3 p02 1µg DOX
SOX9 (~70kDa)
M19
HNO223 wild type
Non-Target p02
shSOX9#1 p02 500ng DOX
shSOX9#1 p02 1µg DOX
Non-Target p02
shSOX9#2 p02 500ng DOX
shSOX9#2 p02 1µg DOX
Non-Target p02
shSOX9#3 p02 500ng DOX
shSOX9#3 p02 1µg DOX
GAPDH (~37kDa)
M20
HNO223 wild type
Non-Target p02
shSOX9#1 p02 500ng DOX
shSOX9#1 p02 1µg DOX
Non-Target p02
shSOX9#2 p02 500ng DOX
shSOX9#2 p02 1µg DOX
Non-Target p02
shSOX9#3 p02 500ng DOX
shSOX9#3 p02 1µg DOX
SOX9 (~70kDa)
M20
HNO223 wild type
Non-Target p02
shSOX9#1 p02 500ng DOX
shSOX9#1 p02 1µg DOX
Non-Target p02
shSOX9#2 p02 500ng DOX
shSOX9#2 p02 1µg DOX
Non-Target p02
shSOX9#3 p02 500ng DOX
shSOX9#3 p02 1µg DOX
GAPDH (~37kDa)
M21
HNO223 wild type
Non-Target p02
shSOX9#1 p02 500ng DOX
shSOX9#1 p02 1µg DOX
shSOX9#3 p05
Non-Target p02
shSOX9#2 p02 500ng DOX
shSOX9#2 p02 1µg DOX
Non-Target p02
shSOX9#3 p02 500ng DOX
shSOX9#3 p02 1µg DOX
Representative image in main text.
SOX9 (~70kDa)
M21
HNO223 wild type
Non-Target p02
shSOX9#1 p02 500ng DOX
shSOX9#1 p02 1µg DOX
Non-Target p02
shSOX9#2 p02 500ng DOX
shSOX9#2 p02 1µg DOX
Non-Target p02
shSOX9#3 p02 500ng DOX
shSOX9#3 p02 1µg DOX
GAPDH (~37kDa)

Representative image in main text.

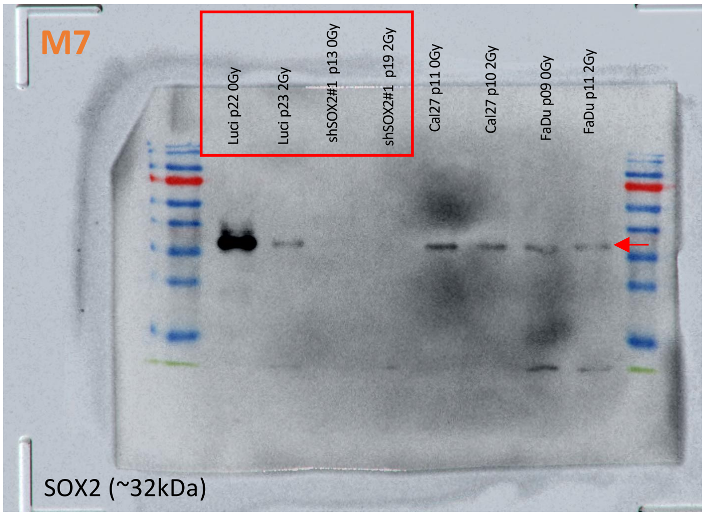

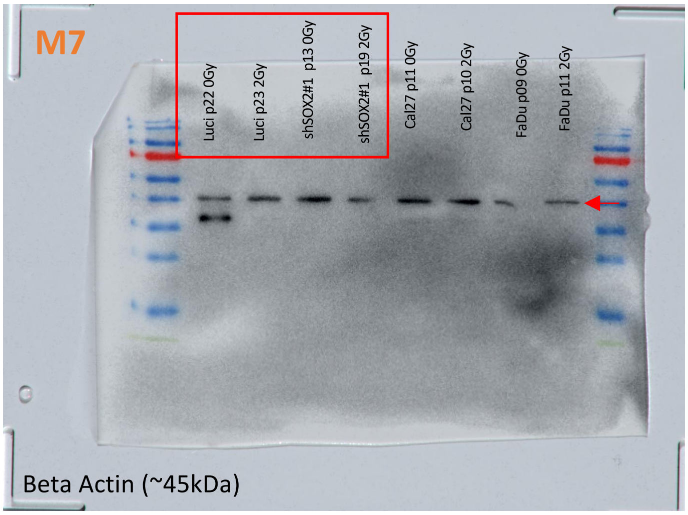

Representative image in main text.

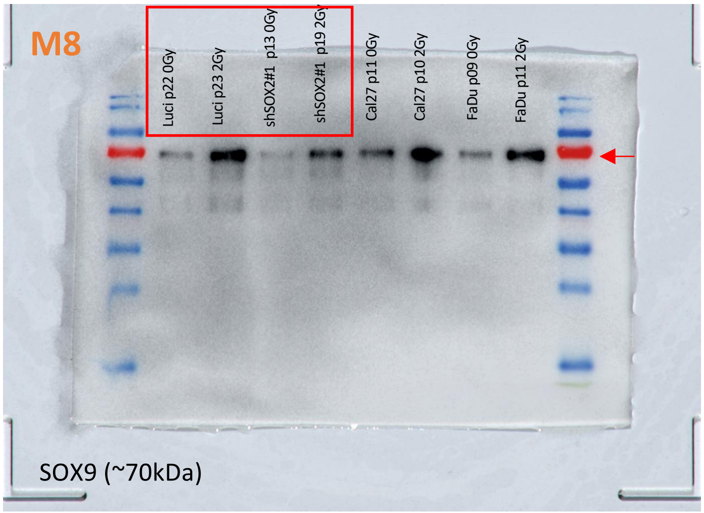

Representative image in main text.

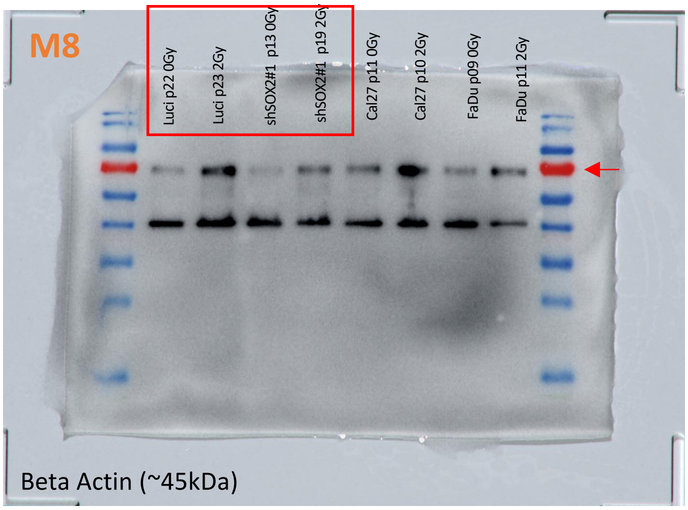

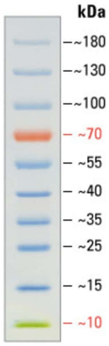

PageRulerTM Prestained Protein Ladder (Thermo Fisher #26616)

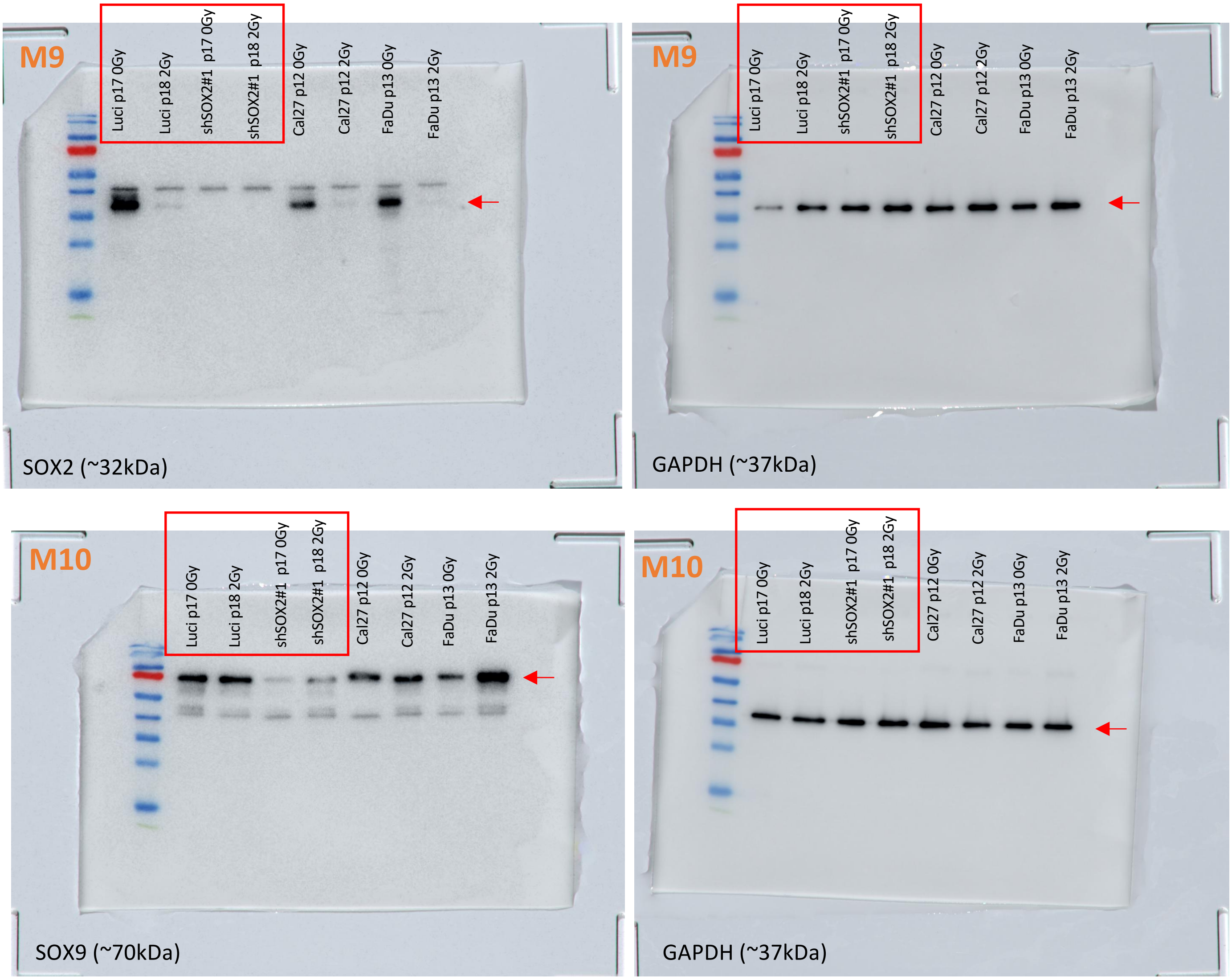

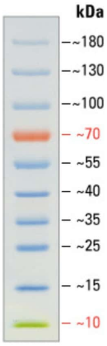

PageRulerTM Prestained Protein Ladder (Thermo Fisher #26616)

Note: For this set of membranes, the loading control/housekeeping gene was first incubated. After washing with 0.1% PBS-T, the target gene (SOX2 or SOX9) was applied to the same membrane.

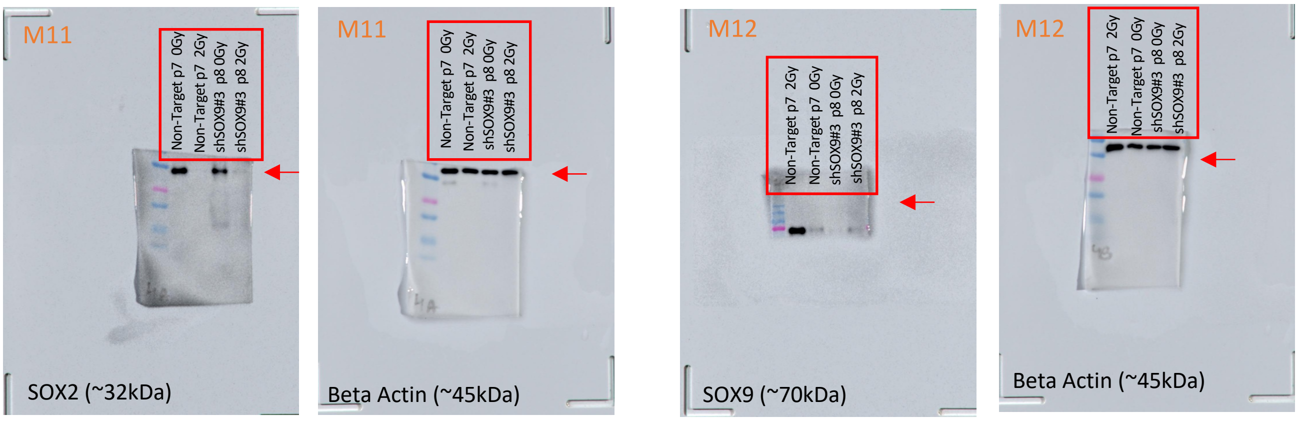

Note:

Equal amount of the same pool of protein was loaded in the same gel in duplicate and after transferred to PVDF membrane. At the end, the membrane was divided where the SOX2 part was initially incubated against SOX2 antibody and later with the loading control (number 11). For SOX9 part we have divide again the membrane and incubated the upper part against SOX9 antibody and the lower part with loading control (number 12).

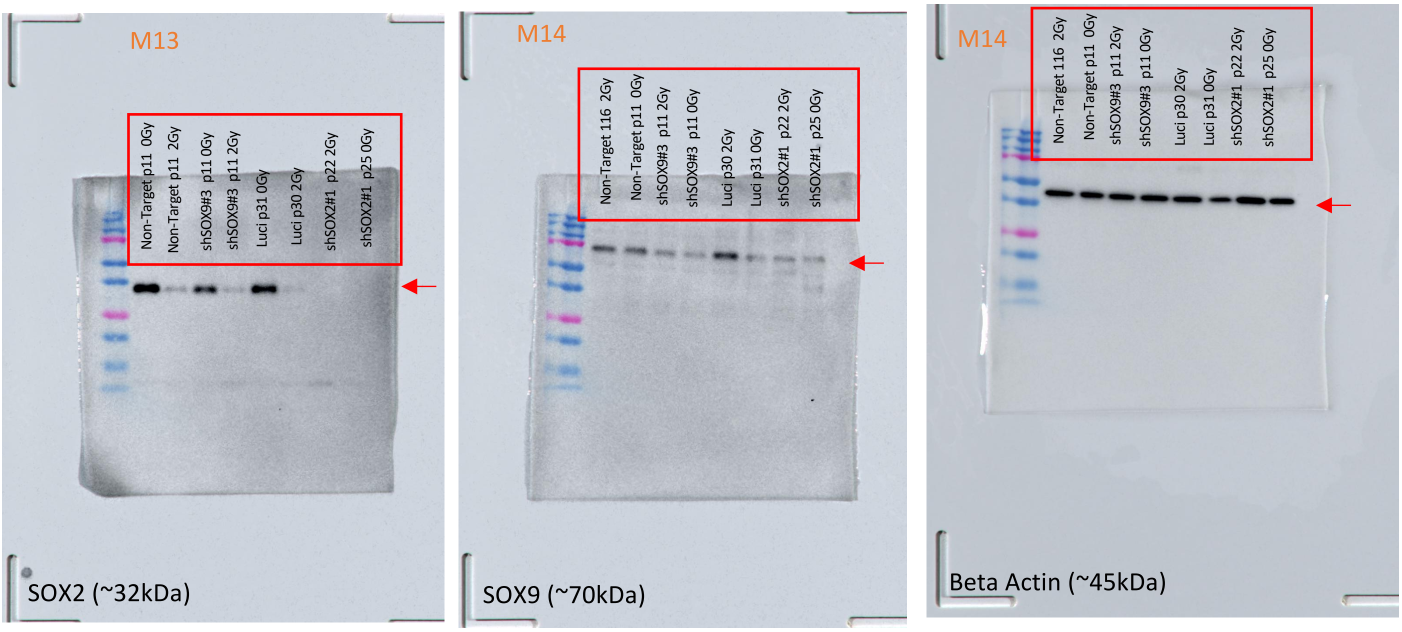

Note:

1. Equal amount of the same pool of protein was loaded in the two gels and after transferred to PVDF membrane. All the conditions were the same and kept constant during all the experimental procedure.

2. The incubation of the loading control was done on the SOX9 membrane as the SOX2 membrane was used for analysis of a third marker.

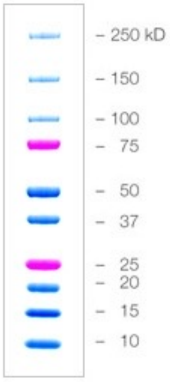

Precision Plus Protein™ Standards Ladder *(BioRad #161-0374)*

Representative image in main text.

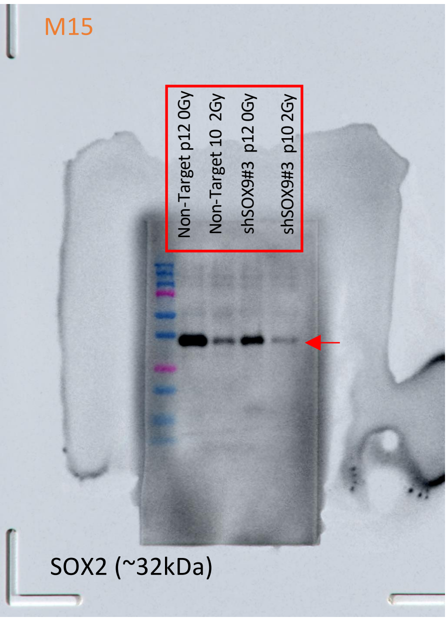

Representative image in main text.

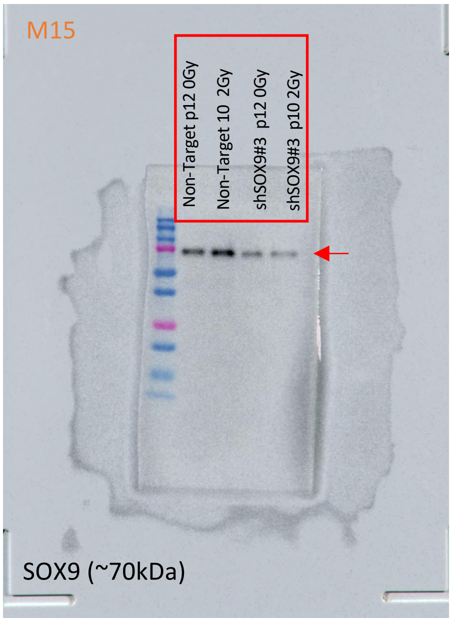

Representative image in main text.

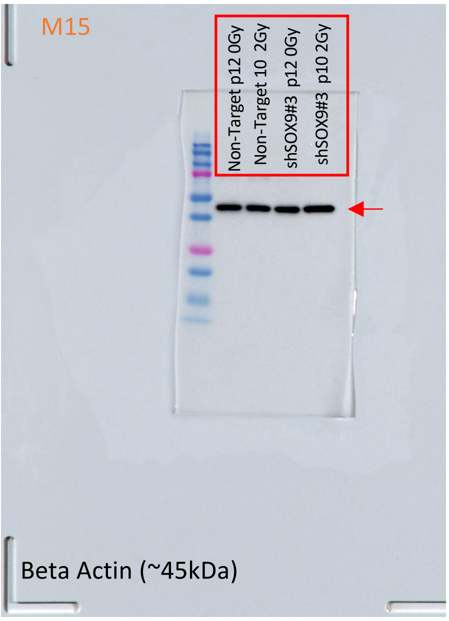

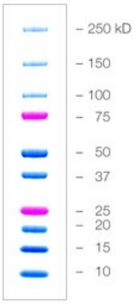

Precision Plus Protein™ Standards Ladder *(BioRad #161-0374)*

Notes:

1. Equal amount of the same pool of protein was loaded in one gel in duplicate and after transferred to PVDF membrane. At the end, the membrane was divided and incubated with the primary antibody against SOX2 or SOX9.

2. The incubation of the loading control was done on the SOX9 membrane as the SOX2 membrane was used for analysis of a third marker.
