# Supplementary material for: The Role of SOX2 and SOX9 in Radioresistance and Tumor Recurrence
Source: Cancers (Basel). 2024 Jan 19;16(2):439. doi: 10.3390/cancers16020439 (PMC10814462; doi:10.3390/cancers16020439)
Supplement: Supplementary file 1 [file cancers-16-00439-s001.zip › cancers-2776517-supplementary/Supplementary Table S1.pdf]

Correlated Gene	Spearman's Correlation SOX2	p-Value	Spearman's Correlation SOX9	p-Value
**UGT1A7**	0,672168643	1,12530E-30	-0,2024429	0,002384367
**ALDH3A1**	0,670920841	1,57961E-30	-0,2828023	1,80900E-05
**UGT1A8**	0,559019725	9,90972E-20	-0,2185055	0,001021912
**AKR1C3**	0,508214372	4,77727E-16	-0,2075712	0,001831420
**NPL**	0,48615644	1,24837E-14	-0,2383943	0,000328270
**KHK**	0,450784104	1,46783E-12	-0,2184638	0,001024243
**GSTM2**	0,448848192	1,87606E-12	-0,2404763	0,000289831
**SLC25A48**	0,436236069	8,93940E-12	-0,2153006	0,001216088
**KEAP1**	0,428427507	2,27675E-11	-0,2046873	0,002125960
**ABCB6**	0,424134737	3,76818E-11	-0,3061501	3,18606E-06
**HOGA1**	0,418008486	7,64027E-11	-0,235814	0,000382479
**EVPLL**	0,414120911	1,18774E-10	-0,267538	5,19325E-05
**SERPINI1**	0,401966428	4,55159E-10	-0,2483465	0,000179227
**GCHFR**	0,401024984	5,03943E-10	-0,24071	0,000285788
**OTOP3**	0,392911286	1,19613E-09	-0,2396772	0,000304060
**SEMA3G**	0,389681121	1,67657E-09	-0,2073082	0,001856646
**METTL7A**	0,383728379	3,09414E-09	-0,2112125	0,001512818
**ARMC7**	0,381863887	3,73942E-09	-0,2710743	4,09019E-05
**SMAD5-AS1**	0,371651921	1,03346E-08	-0,2558629	0,000111596
**ADAD2**	0,370934688	1,10849E-08	-0,2233062	0,000783834
**PRDX2**	0,365645073	1,84896E-08	-0,2419263	0,000265581
**CYP4F12**	0,363667501	2,23342E-08	-0,2156144	0,001195679
**AHRR**	0,359295194	3,37603E-08	-0,2164954	0,001140052
**MIR9-3HG**	0,351908643	6,69073E-08	-0,2441382	0,000232203
**EXPH5**	0,349985716	7,97207E-08	-0,2089271	0,001706244
**ATPAF1**	0,343850096	1,38359E-07	-0,2481972	0,000180895
**PANK1**	0,336018794	2,74941E-07	-0,2140693	0,001299296
**NKD2**	0,330143965	4,54590E-07	-0,2148993	0,001242657
**SLC48A1**	0,32906401	4,98048E-07	-0,2667869	5,46107E-05
**LCMT2**	0,326787229	6,03067E-07	-0,2615397	7,72768E-05
**ACSS2**	0,325718095	6,59406E-07	-0,2679783	5,04203E-05
**REEP1**	0,313048635	1,85187E-06	-0,2577848	9,86333E-05
**ADI1**	0,309383495	2,47484E-06	-0,3422258	1,59786E-07
**CBFA2T3**	0,300567464	4,89350E-06	-0,2360969	0,000376153
**CBR1**	0,300473319	4,92867E-06	-0,2136397	0,001329533
**PCBD2**	0,298995143	5,51336E-06	-0,2092192	0,001680316
**MLYCD**	0,293147367	8,53911E-06	-0,2625061	7,25302E-05
**GABRQ**	0,291363532	9,73969E-06	-0,2143868	0,001277352
**DCXR**	0,290702859	1,02237E-05	-0,2707302	4,18695E-05
**SHMT1**	0,28984582	1,08856E-05	-0,2327846	0,000456689
**POMGNT2**	0,287249814	1,31474E-05	-0,2004681	0,002634997
**RPP25**	0,28642091	1,39589E-05	-0,2149739	0,001237674

**SFXN2**	0,282614012	1,83338E-05	-0,2069392	0,001892579
**FBXL22**	0,282319676	1,87213E-05	-0,2543068	0,000123243
**SULT1A1**	0,281650925	1,96307E-05	-0,2000558	0,002690250
**SLC25A10**	0,279528888	2,28008E-05	-0,2352507	0,000395369
**GSTA3**	0,277254233	2,67332E-05	-0,2244243	0,000736286
**PROS1**	0,265582527	5,91783E-05	-0,2300944	0,000533557
**KAT14**	0,263089323	6,97992E-05	-0,2385447	0,000325342
**MCUR1**	0,262756642	7,13448E-05	-0,2183508	0,001030589
**MYCL**	0,26258289	7,21648E-05	-0,232382	0,000467498
**TSGA10IP**	0,261158532	7,92283E-05	-0,2639299	6,60330E-05
**LDHD**	0,256726457	0,000105585	-0,2474549	0,000189407
**SPIN2A**	0,248888587	0,000173291	-0,2047556	0,002118517
**ZNF503-AS2**	0,24110281	0,000279110	-0,2000407	0,002692301
**PDZD4**	0,237965744	0,000336748	-0,2217448	0,000854979
**ZNF540**	0,236796613	0,000360924	-0,2253591	0,000698590
**ADAL**	0,233998693	0,000425476	-0,20525	0,002065307
**SELENOP**	0,231711091	0,000486041	-0,2045942	0,002136147
**KRT4**	0,226189636	0,000666603	-0,2177783	0,001063285
**CYP1A2**	0,221491903	0,000867045	-0,2519494	0,000143074
**MPC1**	0,22112363	0,000884898	-0,2228886	0,000802307
**USH1G**	0,220489567	0,000916433	-0,2001132	0,002682494
**HADH**	0,216987768	0,001110008	-0,2118142	0,001465347
**BRCC3**	0,216245433	0,001155590	-0,2066178	0,001924391
**VARS2**	0,215755233	0,001186625	-0,2435159	0,000241174
**GPR55**	0,214783006	0,001250452	-0,2434846	0,000241634
**CCDC152**	0,213959996	0,001306928	-0,246139	0,000205419
**STX19**	0,208639269	0,001732140	-0,2098539	0,001625226
**PDIA2**	0,205545596	0,002034082	-0,2086775	0,001728682
**NECAB3**	0,201245087	0,002533664	-0,234846	0,000404878
**AVPR2**	0,200891513	0,002579331	-0,2628889	7,07264E-05
**ACTG1**	-0,200351256	0,002650554	0,26322783	6,91650E-05
**PLEKHG2**	-0,200494096	0,002631552	0,23151956	0,000491458
**RHBDF1**	-0,201278633	0,002529370	0,20189761	0,00245131
**KLF7**	-0,2023034	0,002401330	0,22888027	0,00057203
**LINC00452**	-0,203031215	0,002314003	0,2327531	0,000457526
**EZR**	-0,203661461	0,002240727	0,20667733	0,001918463
**NFIL3**	-0,206202279	0,001966245	0,35167274	6,837E-08
**CHI3L2**	-0,208849144	0,001713220	0,20012293	0,002681188
**CSNK1E**	-0,214851663	0,001245844	0,26231344	7,34539E-05
**WWC2**	-0,214922001	0,001241138	0,20168659	0,002477672
**MARK3**	-0,218930173	0,000998437	0,20611354	0,001975289
**UBASH3B**	-0,220984037	0,000891754	0,32530364	6,82572E-07
**GMEB1**	-0,222990287	0,000797771	0,22984227	0,000541344

**LTBR**	-0,224643766	0,000727271	0,2041192	0,002188839
**LRRC8C**	-0,225342816	0,000699232	0,28950279	1,11618E-05
**ADAMTS6**	-0,22655918	0,000652810	0,2278534	0,000606554
**KDSR**	-0,228802353	0,000574584	0,20295808	0,002322645
**LASP1**	-0,230940622	0,000508174	0,22900146	0,000568077
**VIM**	-0,231811728	0,000483217	0,22536879	0,000698210
**C3ORF52**	-0,234749684	0,000407171	0,2991553	5,44696E-06
**PPP1R14C**	-0,235664075	0,000385871	0,27685172	2,74926E-05
**GPRC5A**	-0,237015643	0,000356275	0,22153592	0,000864933
**CHST15**	-0,237321883	0,000349869	0,23971229	0,000303421
**LAMC2**	-0,244683545	0,000224596	0,25939497	8,88741E-05
**CDCP1**	-0,245683424	0,000211251	0,23538164	0,000392337
**SLC20A1**	-0,246153064	0,000205241	0,33995446	1,95158E-07
**THBS1**	-0,246403033	0,000202108	0,2160052	0,001170705
**TES**	-0,248531563	0,000177179	0,26338799	6,84384E-05
**RP1**	-0,248857616	0,000173625	0,251604	0,000146219
**LINC01119**	-0,252720723	0,000136279	0,21180464	0,001466086
**ITGB6**	-0,252774555	0,000135816	0,24926849	0,000169240
**APCDD1L**	-0,254850061	0,000119053	0,24670494	0,000198383
**FOXC2**	-0,256316334	0,000108401	0,26477094	6,24563E-05
**CTSV**	-0,256497048	0,000107152	0,23835855	0,000328969
**ABL2**	-0,258186237	9,61101E-05	0,32613038	6,37108E-07
**ICAM1**	-0,260477085	8,28326E-05	0,2875777	1,28390E-05
**DCBLD2**	-0,261329796	7,83459E-05	0,30619773	3,17430E-06
**ADRA1B**	-0,262021885	7,48731E-05	0,31945888	1,10477E-06
**ELFN2**	-0,263208684	6,92523E-05	0,24364972	0,000239218
**PTPRE**	-0,264347828	6,42322E-05	0,21000701	0,001612190
**ENC1**	-0,264367306	6,41494E-05	0,33537926	2,90559E-07
**PPP4R4**	-0,264571898	6,32859E-05	0,26919796	4,64454E-05
**SLC35F2**	-0,265129119	6,09891E-05	0,21903838	0,000992536
**MCL1**	-0,269443531	4,56816E-05	0,22736746	0,000623554
**GFPT2**	-0,276619068	2,79408E-05	0,27854416	2,44309E-05
**TINAGL1**	-0,276828999	2,75361E-05	0,3443706	1,32097E-07
**LTBP1**	-0,282466844	1,85266E-05	0,23198811	0,000478303
**TMCC3**	-0,28461377	1,58954E-05	0,27715147	2,69252E-05
**COL13A1**	-0,287433774	1,29735E-05	0,22834462	0,000589805
**SRPX**	-0,2899075	1,08367E-05	0,23083674	0,000511229
**FRMD5**	-0,292728733	8,80755E-06	0,21861308	0,001015917
**FOXD1**	-0,293141957	8,54253E-06	0,22280957	0,000805846
**MED15**	-0,293824774	8,12112E-06	0,21843889	0,001025638
**MYADM**	-0,298066685	5,91371E-06	0,21042255	0,001577281
**LITAF**	-0,307841474	2,79273E-06	0,30263972	4,17724E-06
**MICAL2**	-0,308202902	2,71491E-06	0,260476	8,28385E-05

**TENM3**	-0,313222856	1,82634E-06	0,23100122	0,000506400
**DNAJB5**	-0,31427143	1,67965E-06	0,23373033	0,000432199
**PCDHGC5**	-0,315364996	1,53867E-06	0,21108919	0,001522718
**MANCR**	-0,316318061	1,42509E-06	0,21144213	0,001494536
**C1ORF74**	-0,31735439	1,31068E-06	0,25437176	0,000122735
**PDE7A**	-0,317529693	1,29222E-06	0,2343612	0,000416547
**C10ORF55**	-0,31762492	1,28229E-06	0,26364229	6,72995E-05
**ST3GAL5**	-0,319606281	1,09157E-06	0,2340085	0,000425232
**TIMP3**	-0,320916729	9,80666E-07	0,23793328	0,000337398
**PLAU**	-0,325032031	6,98175E-07	0,27620786	2,87499E-05
**SERPINB7**	-0,33058006	4,38092E-07	0,22849828	0,000584654
**CCDC71L**	-0,332492165	3,72287E-07	0,22186164	0,000849454
**SEMA7A**	-0,333543986	3,40242E-07	0,27988491	2,22371E-05
**GNB1**	-0,333696565	3,35819E-07	0,20364306	0,002242835
**TRAF3IP2**	-0,333795038	3,32994E-07	0,29019426	1,06117E-05
**PMEPA1**	-0,33634343	2,67325E-07	0,24424745	0,000230660
**FOSL1**	-0,337122557	2,49863E-07	0,21403574	0,001301634
**TREM1**	-0,337424468	2,43395E-07	0,2089606	0,001703249
**NME7**	-0,344884603	1,26181E-07	0,22195578	0,000845028
**TTPAL**	-0,353500442	5,78222E-08	0,21787186	0,001057878
**CGB8**	-0,354312725	5,36560E-08	0,21392522	0,001309365
**SH3KBP1**	-0,354525209	5,26147E-08	0,32235703	8,71217E-07
**RTTN**	-0,355802111	4,67555E-08	0,21544358	0,001206752
**PRDM8**	-0,357313832	4,06289E-08	0,27024863	4,32597E-05
**MSN**	-0,358283412	3,71147E-08	0,22347508	0,000776474
**TENM3-AS1**	-0,360546124	3,00154E-08	0,30489919	3,51033E-06
**SERPINE1**	-0,361473501	2,75009E-08	0,37570121	6,93515E-09
**DUSP6**	-0,361817615	2,66205E-08	0,26487698	6,20185E-05
**DUSP4**	-0,365217636	1,92622E-08	0,29544795	7,19717E-06
**MRTFA**	-0,366096317	1,77063E-08	0,27528481	3,06473E-05
**BACE2**	-0,368057119	1,46590E-08	0,23511977	0,000398422
**PLAUR**	-0,36872164	1,37461E-08	0,24709349	0,000193684
**CITED2**	-0,369365402	1,29142E-08	0,20930363	0,001672893
**DYRK3**	-0,369488763	1,27605E-08	0,26923576	4,63271E-05
**MAP3K20**	-0,370114229	1,20077E-08	0,25920018	9,00045E-05
**STK17A**	-0,370199716	1,19082E-08	0,20004718	0,002691422
**PLK3**	-0,37511254	7,35172E-09	0,29658526	6,61025E-06
**CHST11**	-0,377114462	6,02604E-09	0,31210394	1,99633E-06
**ADTRP**	-0,378122998	5,44885E-09	0,20897359	0,001702091
**ERRFI1**	-0,378416253	5,29130E-09	0,32001099	1,05610E-06
**RPSAP52**	-0,383618418	3,12901E-09	0,29882774	5,58358E-06
**NOX5**	-0,387888514	2,01893E-09	0,2886696	1,18604E-05
**ARHGAP29**	-0,3882182	1,95126E-09	0,28770222	1,27236E-05

**INHBA**	-0,39146229	1,39237E-09	0,29617297	6,81755E-06
**EVA1A**	-0,392218692	1,28634E-09	0,29486253	7,51825E-06
**PPP1R18**	-0,396411907	8,26151E-10	0,21097876	0,001531636
**CANT1**	-0,400063088	5,59007E-10	0,26049393	8,27417E-05
**SMIM3**	-0,406374985	2,81353E-10	0,24635975	0,000202647
**IER5**	-0,40728613	2,54503E-10	0,2193338	0,000976588
**ANXA5**	-0,412270374	1,46242E-10	0,22577458	0,000682416
**L1CAM**	-0,412718372	1,39075E-10	0,22361251	0,000770533
**ITGA5**	-0,416788225	8,78048E-11	0,29306729	8,58985E-06
**ACTN1**	-0,425553396	3,19271E-11	0,32693223	5,95790E-07
**CAMK2N1**	-0,427583453	2,51527E-11	0,27860259	2,43311E-05
**DCBLD1**	-0,430887165	1,70037E-11	0,33774153	2,36776E-07
**SH3TC2**	-0,431979682	1,49247E-11	0,2340085	0,000425232
**SEC14L2**	-0,433178013	1,29286E-11	0,39166356	1,36336E-09
**KLF6**	-0,440251831	5,47562E-12	0,23558941	0,000387571
**ANTXR2**	-0,440625162	5,22999E-12	0,23222402	0,000471805
**INPP4B**	-0,444617103	3,19037E-12	0,23803825	0,000335299
**MICALCL**	-0,457940163	5,84581E-13	0,2120717	0,001445446
**KIF12**	-0,458160498	5,68049E-13	0,22043871	0,000919006
**TNS4**	-0,460269967	4,31169E-13	0,20441245	0,002156173
**CCBE1**	-0,461885697	3,48641E-13	0,21624928	0,001155349
**CEBPB**	-0,464469675	2,47630E-13	0,24036264	0,000291816
**LGALS1**	-0,464565983	2,44478E-13	0,24179537	0,00026769
**CDH3**	-0,469836989	1,20550E-13	0,2328365	0,000455312
**TNFRSF12A**	-0,497349888	2,45440E-15	0,29019426	1,06117E-05
**TRIML2**	-0,498719069	2,00335E-15	0,20544101	0,002045081
**TMEM51**	-0,516323779	1,35499E-16	0,27402631	3,34252E-05
**FHL2**	-0,547325432	7,93786E-19	0,22600074	0,000673757
**BCAR3**	-0,549906288	5,05019E-19	0,27681926	2,75547E-05
**TMEM92**	-0,586926693	4,892938E-22	0,22461996	0,000728244
