# Supplementary material for: The Role of SOX2 and SOX9 in Radioresistance and Tumor Recurrence
Source: Cancers (Basel). 2024 Jan 19;16(2):439. doi: 10.3390/cancers16020439 (PMC10814462; doi:10.3390/cancers16020439)
Supplement: Supplementary file 1 [file cancers-16-00439-s001.zip › cancers-2776517-supplementary/Supplementary Table S2.pdf]

Gene Name	Gene description	Category	log2FC	lfcSE	padj	Spearman's Correlation SOX2	Spearman's Correlation SOX9	SOX_Group
ADAD2	Adenosine Deaminase Domain Containing 2	Protein coding	-1,158	0,186	0,000	0,371	-0,223	SOX2HighSOX9Low
AKR1C3	Aldo-Keto Reductase Family 1 Member C3	Protein coding	-2,298	0,291	0,000	0,508	-0,208	SOX2HighSOX9Low
ALDH3A1	Aldehyde Dehydrogenase 3 Family Member A1	Protein coding	-2,712	0,317	0,000	0,671	-0,283	SOX2HighSOX9Low
EVPLL	Envoplakin Like	Protein coding	-1,598	0,284	0,000	0,414	-0,268	SOX2HighSOX9Low
GCHFR	GTP Cyclohydrolase	Protein coding	-1,202	0,166	0,000	0,401	-0,241	SOX2HighSOX9Low
GSTA3	Glutathione S-Transferase Alpha 3	Protein coding	-1,746	0,465	0,000	0,277	-0,224	SOX2HighSOX9Low
GSTM2	Glutathione S-Transferase Mu 2	Protein coding	-2,486	0,252	0,000	0,449	-0,240	SOX2HighSOX9Low
HOGA1	4-Hydroxy-2-Oxoglutarate Aldolase 1	Protein coding	-1,187	0,216	0,000	0,418	-0,236	SOX2HighSOX9Low
MIR9-3HG	MIR9-3 Host Gene	RNA gene	-1,195	0,246	0,000	0,352	-0,24	SOX2HighSOX9Low
NKD2	NKD Inhibitor Of WNT Signaling Pathway 2	Protein coding	-1,297	0,184	0,000	0,330	-0,215	SOX2HighSOX9Low
PDIA2	Protein Disulfide Isomerase Family A Member 2	Protein coding	-1,247	0,413	0,002	0,206	-0,209	SOX2HighSOX9Low
SERPINI1	Serpin Family I Member 1	Protein coding	-2,606	0,235	0,000	0,402	-0,248	SOX2HighSOX9Low
SLC25A48	Solute Carrier Family 25 Member 48	Protein coding	-2,090	0,302	0,000	0,436	-0,215	SOX2HighSOX9Low
TSGA10IP	Testis Specific 10 Interacting Protein	Protein coding	-2,339	0,375	0,000	0,261	-0,264	SOX2HighSOX9Low
UGT1A7	UDP Glucuronosyltransferase Family 1 Member A7	Protein coding	-4,791	0,437	0,000	0,672	-0,202	SOX2HighSOX9Low
UGT1A8	UDP Glucuronosyltransferase Family 1 Member A8	Protein coding	-4,877	0,595	0,000	0,559	-0,219	SOX2HighSOX9Low
ACTN1	Actinin Alpha 1	Protein coding	1,112	0,124	0,000	-0,426	0,327	SOX2LowSOX9High
ADRA1B	Adrenoceptor Alpha 1B	Protein coding	1,191	0,300	0,000	-0,262	0,319	SOX2LowSOX9High
ADTRP	Androgen Dependent TFPI Regulating Protein	Protein coding	1,525	0,204	0,000	-0,378	0,209	SOX2LowSOX9High
ANTXR2	ANTXR Cell Adhesion Molecule 2	Protein coding	1,166	0,172	0,000	-0,441	0,232	SOX2LowSOX9High
APCDD1L	APC Down-Regulated 1 Like	Protein coding	1,458	0,264	0,000	-0,255	0,247	SOX2LowSOX9High
ARHGAP29	Rho GTPase Activating Protein 29	Protein coding	1,007	0,171	0,000	-0,388	0,288	SOX2LowSOX9High
BCAR3	BCAR3 Adaptor Protein, NSP Family Member	Protein coding	1,498	0,157	0,000	-0,550	0,277	SOX2LowSOX9High

CAMK2N1	Calcium/Calmodulin Dependent Protein Kinase II Inhibitor 1	Protein coding	1,017	0,188	0,000	-0,428	0,279	SOX2LowSOX9High
CCBE1	Collagen And Calcium Binding EGF Domains 1	Protein coding	1,952	0,265	0,000	-0,462	0,216	SOX2LowSOX9High
CDH3	Cadherin 3	Protein coding	1,253	0,128	0,000	-0,470	0,233	SOX2LowSOX9High
CEBPB	CCAAT Enhancer Binding Protein Beta	Protein coding	1,136	0,114	0,000	-0,464	0,240	SOX2LowSOX9High
CGB8	Chorionic Gonadotropin Subunit Beta 8	Protein coding	2,811	0,420	0,000	-0,354	0,214	SOX2LowSOX9High
CHI3L2	Chitinase 3 Like 2	Protein coding	1,402	0,260	0,000	-0,209	0,200	SOX2LowSOX9High
DCBLD1	Discoidin, CUB And LCCL Domain Containing 1	Protein coding	1,291	0,134	0,000	-0,431	0,338	SOX2LowSOX9High
DUSP4	Dual Specificity Phosphatase 4	Protein coding	1,085	0,194	0,000	-0,365	0,295	SOX2LowSOX9High
DUSP6	Dual Specificity Phosphatase 6	Protein coding	1,338	0,150	0,000	-0,362	0,265	SOX2LowSOX9High
ELFN2	Extracellular Leucine Rich Repeat And Fibronectin Type III Domain Containing 2	Protein coding	2,042	0,334	0,000	-0,263	0,244	SOX2LowSOX9High
EVA1A	Eva-1 Homolog A, Regulator Of Programmed Cell Death	Protein coding	1,371	0,222	0,000	-0,392	0,295	SOX2LowSOX9High
FHL2	Four And A Half LIM Domains 2	Protein coding	1,333	0,122	0,000	-0,547	0,226	SOX2LowSOX9High
FOSL1	FOS Like 1, AP-1 Transcription Factor Subunit	Protein coding	1,041	0,177	0,000	-0,337	0,214	SOX2LowSOX9High
FOXC2	Forkhead Box C2	Protein coding	2,064	0,249	0,000	-0,256	0,265	SOX2LowSOX9High
FRMD5	FERM Domain Containing 5	Protein coding	1,008	0,259	0,000	-0,293	0,219	SOX2LowSOX9High
**INHBA**	Inhibin Subunit Beta A	Protein coding	1,609	0,218	0,000	-0,391	0,296	SOX2LowSOX9High
INPP4B	Inositol Polyphosphate-4-Phosphatase Type II B	Protein coding	1,417	0,179	0,000	-0,445	0,238	SOX2LowSOX9High
**ITGB6**	Integrin Subunit Beta 6	Protein coding	1,392	0,188	0,000	-0,253	0,249	SOX2LowSOX9High
KIF12	Kinesin Family Member 12	Protein coding	1,685	0,271	0,000	-0,458	0,220	SOX2LowSOX9High
L1CAM	L1 Cell Adhesion Molecule	Protein coding	1,488	0,278	0,000	-0,413	0,224	SOX2LowSOX9High
LAMC2	Laminin Subunit Gamma 2	Protein coding	1,285	0,204	0,000	-0,245	0,259	SOX2LowSOX9High
LGALS1	Galectin 1	Protein coding	1,123	0,178	0,000	-0,465	0,242	SOX2LowSOX9High
LINC00452	Long Intergenic Non-Protein Coding RNA 452	RNA gene	2,161	0,391	0,000	-0,203	0,233	SOX2LowSOX9High

**LTBP1**	Latent Transforming Growth Factor Beta Binding Protein 1	Protein coding	1,178	0,174	0,000	-0,282	0,232	SOX2LowSOX9High
MANCR	Mitotically Associated Long Non Coding RNA	RNA gene	1,507	0,265	0,000	-0,316	0,21	SOX2LowSOX9High
NOX5	NADPH Oxidase 5	Protein coding	2,188	0,226	0,000	-0,388	0,289	SOX2LowSOX9High
PCDHGC5	Protocadherin Gamma Subfamily C, 5	Protein coding	2,029	0,324	0,000	-0,315	0,211	SOX2LowSOX9High
PMEPA1	Prostate Transmembrane Protein, Androgen Induced 1	Protein coding	1,247	0,167	0,000	-0,336	0,244	SOX2LowSOX9High
PPP4R4	Protein Phosphatase 4 Regulatory Subunit 4	Protein coding	1,416	0,242	0,000	-0,265	0,269	SOX2LowSOX9High
PRDM8	PR/SET Domain 8	Protein coding	1,120	0,163	0,000	-0,357	0,270	SOX2LowSOX9High
RP1	RP1 Axonemal Microtubule Associated	Protein coding	1,723	0,306	0,000	-0,249	0,252	SOX2LowSOX9High
RPSAP52	Ribosomal Protein SA Pseudogene 52	Pseudogene	1,927	0,273	0,000	-0,384	0,299	SOX2LowSOX9High
RTTN	Rotatin	Protein coding	1,038	0,153	0,000	-0,356	0,215	SOX2LowSOX9High
SEC14L2	SEC14 Like Lipid Binding 2	Protein coding	1,535	0,169	0,000	-0,433	0,392	SOX2LowSOX9High
SERPINB7	Serpin Family B Member 7	Protein coding	1,747	0,278	0,000	-0,331	0,228	SOX2LowSOX9High
**SERPINE1**	Serpin Family E Member 1	Protein coding	1,543	0,210	0,000	-0,361	0,376	SOX2LowSOX9High
SH3TC2	SH3 Domain And Tetratricopeptide Repeats 2	Protein coding	1,219	0,189	0,000	-0,432	0,234	SOX2LowSOX9High
SRPX	Sushi Repeat Containing Protein X-Linked	Protein coding	1,024	0,226	0,000	-0,290	0,231	SOX2LowSOX9High
TENM3	Teneurin Transmembrane Protein 3	Protein coding	1,071	0,222	0,000	-0,313	0,231	SOX2LowSOX9High
**THBS1**	Thrombospondin 1	Protein coding	1,120	0,212	0,000	-0,246	0,216	SOX2LowSOX9High
TINAGL1	Tubulointerstitial Nephritis Antigen Like 1	Protein coding	1,253	0,177	0,000	-0,277	0,344	SOX2LowSOX9High
TMEM92	Transmembrane Protein 92	Protein coding	1,716	0,187	0,000	-0,587	0,225	SOX2LowSOX9High
TNFRSF12A	TNF Receptor Superfamily Member 12A	Protein coding	1,287	0,129	0,000	-0,497	0,290	SOX2LowSOX9High
TNS4	Tensin 4	Protein coding	1,070	0,142	0,000	-0,460	0,204	SOX2LowSOX9High
TREM1	Triggering Receptor Expressed On Myeloid Cells 1	Protein coding	1,069	0,245	0,000	-0,337	0,209	SOX2LowSOX9High
TRIML2	Tripartite Motif Family Like 2	Protein coding	3,328	0,405	0,000	-0,499	0,205	SOX2LowSOX9High
