# Supplementary material for: The Role of SOX2 and SOX9 in Radioresistance and Tumor Recurrence
Source: Cancers (Basel). 2024 Jan 19;16(2):439. doi: 10.3390/cancers16020439 (PMC10814462; doi:10.3390/cancers16020439)
Supplement: Supplementary file 1 [file cancers-16-00439-s001.zip › cancers-2776517-supplementary/Supplementary Table S3.pdf]

Ingenuity Canonical Pathways	-log(p-value)	Molecules	Key Biological Function
TGF-β Signaling	2,500	**INHBA**, PMEPA1, **SERPINE1**	Cell migration, cell differentiation, apoptosis, cell growth, etc.
Integrin Signaling	1,610	**ACTN1**, BCAR3, **ITGB6**	Promote intracellular signals in response to the stimmulus by the extracellular matrix resulting in modification in cellular shape, mobility, and progression through the cell cycle.
NRF2-mediated Oxidative Stress Response	1,570	FOSL1, GSTA3, GSTM2	It is activated under oxidative stress conditions and subsequently activates several antioxidative genes and proteins.
ERK/MAPK Signaling	1,550	DUSP4, DUSP6, **ITGB6**	It is a chain of proteins in the cell that communicates a signal from a receptor on the surface of the cell to the DNA in the nucleus of the cell.
Paxillin Signaling	1,380	**ACTN1, ITGB6**	Focal adhesions and it functions to adhere cells to the extracellular matrix.
TWEAK Signaling	0,983	TNFRSF12A	It is related to TNF and presented apoptotic ability
IL-17A Signaling in Fibroblasts	0,959	**CEBPB**	Play a central role in the control of infections and chronic inflammatory conditions
ILK Signaling	0,921	**ACTN1, ITGB6**	Control of cytoskeletal organization and cell motility.
HIF1-alpha Signaling	0,889	ADRA1B, **SERPINE1**	Master transcriptional regulator of cellular and developmental response to hypoxia
RHOGDI Signaling	0,863	CDH3, **ITGB6**	Regulates cell migration, adhesion, proliferation and differentiation.
Transcriptional Regulatory Network in Embryonic Stem Cells	0,818	L1CAM	Process that modulate cellular idenity and modulates biological activity.
cAMP-mediated signaling	0,804	DUSP4, DUSP6	Signaling cascade used in cell communication.
Wound Healing Signaling Pathway	0,785	**CEBPB**, LAMC2	Process of tissue regeneration and repair.
Actin Cytoskeleton Signaling	0,777	**ACTN1, ITGB6**	Mediate cell motility and cell shape changes during the cell cycle and in response to extracellular stimulus
Eicosanoid Signaling	0,742	AKR1C3	Cell signaling, innune response, cell growth, inflamation, etc.
Remodeling of Epithelial Adherens Junctions	0,738	**ACTN1**	Principle mediators of cell-cell adhesion
Signaling by Rho Family GTPases	0,712	**CDH3, ITGB6**	Rho proteins play a role in organelle development, cytoskeletal dynamics, cell movement and other common cellular functions.
TREM1 Signaling	0,703	TREM1	Modulate the immune response stimulated by pathogen and cytokine receptors like IL-1R.
Senescence Pathway	0,646	**CEBPB**, **SERPINE1**	Process of stable cell cycle arrest that response to various intrinsic and extrinsic stimuli, associated with cellular and molecular changes.
p53 Signaling	0,587	**THBS1**	Controlling cell division and cell death.
Role of Tissue Factor in Cancer	0,527	PDIA2	Expressed by tumor cells and contribute to tumor growth, metastasis and tumor angiogenisis.
PAK Signaling	0,521	**ITGB6**	Activation of MAPK siganling in breast cancer
IL-6 Signaling	0,488	**CEBPB**	Cytokine produced by numerous different cell types and plays a critical role in regulating the acute inflammation and cancer progression.
HGF Signaling	0,480	**ITGB6**	Produced by stromal and mesenchymal cells, and it stimulates epithelial cell proliferation, motility, morphogenesis and angiogenesis.
IL-12 Signaling and Production in Macrophages	0,472	**CEBPB**	Promotes Th1 responses and induces IFNγ production by T and NK cells
Protein Kinase A Signaling	0,470	DUSP4, DUSP6	Play a role in many cellular process, such as: proliferation, cell cycle, metabolism, etc.
RAC Signaling	0,465	**ITGB6**	Control of cell- ell adhesion, cell migration, cell-matrix adhesion, cell cycle, etc.
NAD Signaling Pathway	0,445	**CEBPB**	involved in cell metabolism

PTEN Signaling	0,433	**ITGB6**	Promote the activation of signals from growth factor receptors on the cell surface to transcription factors in the nucleus.
HMGB1 Signaling	0,413	**SERPINE1**	Secreted in the tumor microenvironment leading to cell proliferation, differentiation, mobilization and senescence.
Human Embryonic Stem Cell Pluripotency	0,405	**INHBA**	
G∝q Signaling	0,401	ADRA1B	Signal transduction in cells leadind to RhoA activation, phosphatidylinositol 3-kinase (PI3K) and implications in the regulation of the Akt pathway.
WNT/β-catenin Signaling	0,390	CDH3	Biological processes such as proliferation, apoptosis, differentiation, adhesion, metabolism, gene expression, etc.
IL-17 Signaling	0,374	**CEBPB**	Pro-inflammatory cytokine involved in control infections.
Leukocyte Extravasation Signaling	0,357	ACTN1	Promote the movement of leukocytes out of the vesels and towards the site of tissue damage or infection.
Regulation of the Epithelial-Mesenchymal Transition Pathway	0,350	FOXC2	Biological process in which a non-motile epithelial cell changes to a mesenchymal phenotype with invasive capacities
PI3K/AKT Signaling	0,343	**ITGB6**	Intracellular signaling which promotes cell proliferation and apoptosis, angiogenesis and glucose metabolism.
FAK Signaling	0,332	ADRA1B, BCAR3, **ITGB6**	Plays critical roles in integrin-mediated signal transductions.
HER-2 Signaling in Breast Cancer	0,305	**ITGB6**	Promote cells in the breast to grow and divide at an uncontrolled rate.
AMPK Signaling	0,279	ADRA1B	Play a role as regulator of cellular energy homeostasis.
Sirtuin Signaling Pathway	0,234	DUSP6	Envolved in metabolic regulation.
SAPK/JNK Signaling	0,219	DUSP4	Activated by a variety of environmental stresses, inflammatory cytokines, growth factors which may regulate the activity of multiple transcription factors.
Role of Macrophages, Fibroblasts and Endothelial Cells in Rheumatoid Arthritis	0,206	**CEBPB**	Secretion of cytokines involved in the regulation of angiogenisisand ECM reorganization.
Glucocorticoid Receptor Signaling	0,000	**SERPINE1**	Regilation of gene expression
Molecular Mechanisms of Cancer	0,000	**ITGB6**	Causes by specific DNA damage
CDC42 Signaling	0,000	**ITGB6**	Envolved with Ras-mediated cellular transformation, tumorigenesis, and metastasis.
Phospholipase C Signaling	0,000	**ITGB6**	Actvation of Protein kinase C
TEC Kinase Signaling	0,000	**ITGB6**	Signal transducers that mediate cell-to-cell communication.
T Cell Receptor Signaling	0,000	DUSP6	Resulation of cytokine production, cell survival, proliferation, and differentiation.
