# Supplementary material for: The Role of SOX2 and SOX9 in Radioresistance and Tumor Recurrence
Source: Cancers (Basel). 2024 Jan 19;16(2):439. doi: 10.3390/cancers16020439 (PMC10814462; doi:10.3390/cancers16020439)
Supplement: Supplementary file 1 [file cancers-16-00439-s001.zip › cancers-2776517-supplementary/Supplementary Table S4.pdf]

List of Primers used for Real Time qPCR		
**Gene**	**Forward (5′-3′)**	**Reverse (5′-3′)**
INHBA	ATGACATTGGAAGGAGGGCA	TGGGGACTTTTAGGAAGAGCC
SOX2	GCCGAGTGGAAACTTTTGTCG	GCAGCGTGTACTTATCCTTCTT
SOX9	CGAAAGCGGAGCTCGAAACT	GTTTCCGGGGTTGAAACTGG
Serpine1	TCGAGGTGAACGAGAGTGGCA	AAGGACTGTTCCTGTGGGGTTGT
GAPDH	AGCCTCAAGATCATCAGCAATG	CAGTCTTCTGGGTGGCAGTG
ACTB	TCCCTGGAGAAGAGCTACGA	AGGAAGGAAGGCTGGAAGAG
